# Molecular profiles of tumor contrast enhancement: A radiogenomic analysis in anaplastic gliomas

**DOI:** 10.1002/cam4.1672

**Published:** 2018-08-16

**Authors:** Xing Liu, Yiming Li, Zhiyan Sun, Shaowu Li, Kai Wang, Xing Fan, Yuqing Liu, Lei Wang, Yinyan Wang, Tao Jiang

**Affiliations:** ^1^ Beijing Neurosurgical Institute Capital Medical University Beijing China; ^2^ Department of Neurosurgery Beijing Tiantan Hospital Capital Medical University Beijing China; ^3^ Neurological Imaging Center Beijing Neurosurgical Institute Capital Medical University Beijing China; ^4^ Department of Neuroradiology Beijing Tiantan Hospital Capital Medical University Beijing China; ^5^ Molecular Pathology Center Beijing Neurosurgical Institute Capital Medical University Beijing China

**Keywords:** anaplastic glioma, postcontrast enhancement, radiogenomic analysis, transcriptome

## Abstract

The presence of contrast enhancement (CE) on magnetic resonance (MR) imaging is conventionally regarded as an indicator for tumor malignancy. However, the biological behaviors and molecular mechanism of enhanced tumor are not well illustrated. The aim of this study was to investigate the molecular profiles associated with anaplastic gliomas (AGs) presenting CE on postcontrast T1‐weighted MR imaging. In this retrospective database study, RNA sequencing and MR imaging data of 91 AGs from the Cancer Genome Atlas (TCGA) and 64 from the Chinese Glioma Genome Atlas (CGGA) were collected. Gene set enrichment analysis (GSEA), significant analysis of microarray, generalized linear models, and Least absolute shrinkage and selection operator algorithm were used to explore radiogenomic and prognostic signatures of AG patients. GSEA indicated that angiogenesis and epithelial‐mesenchymal transition were significantly associated with post‐CE. Genes driving immune system response, cell proliferation, and focal adhesions were also significantly enriched. Gene ontology of 237 differential genes indicated consistent results. A 48‐gene signature for CE was identified in TCGA and validated in CGGA dataset (area under the curve = 0.9787). Furthermore, seven genes derived from the CE‐specific signature could stratify AG patients into two subgroups based on overall survival time according to corresponding risk score. Comprehensive analysis of post‐CE and genomic characteristics leads to a better understanding of radiology‐pathology correlations. Our gene signature helps interpret the occurrence of radiological traits and predict clinical outcomes. Additionally, we found nine prognostic quantitative radiomic features of CE and investigated the underlying biological processes of them.

## 1 INTRODUCTION

Gliomas are both the most common and lethal tumors of the central nervous system. Magnetic resonance (MR) imaging, an indispensable approach to tumor diagnosis and treatment monitoring, identifies tumor‐specific behaviors and malignancy;^1^, ^2^ contrast enhancement (CE) seen on MR imaging is indicative of blood‐brain barrier disruption and tumor cells infiltration. Abnormalities in the focal blood‐brain barrier can lead to the leakage of contrast reagents, which results in an enhancement on T1‐weighted images. Moreover, CE has been positively correlated with tumor malignancy and unfavorable prognosis. Almost 90% of glioblastoma (GBMs; World Health Organization [WHO] grade IV) are reportedly enhanced after contrast administration, with a corresponding overall survival time of 14.4 months. Meanwhile, the enhancement ratio and overall survival for low‐grade gliomas (WHO grade II) are only 10% and 78.1 months, respectively.^3^, ^4^

Multi‐omics studies have greatly increased our insight into relationships between genetic alterations and radiographic imaging phenotypes, and a new research field named “radiogenomics” was generated.^5^ A previous study revealed that 1p/19q‐codeleted and CE anaplastic oligodendrogliomas present larger tumor volumes, chromosome 9p and *CDKN2A* loss, genomic instability, and expression of angiogenesis‐related genes.^1^ Another radiogenmic study identified significant imaging correlations for six driver genes both in regions of enhancement and nonenhancing parenchyma.^6^ However, an integrative radiogenomic analysis for clarifying molecular pathways corresponding to CEs in brain tumors have not been conducted yet.

In the present study, we investigated the specific genetic alterations associated with anaplastic gliomas (AGs, WHO grade III) presenting with CE on postcontrast T1‐weighted MR images. Unlike GBM and low‐grade glioma, 62%‐80% of AG patients present with CE, making them suitable subjects to explore radiogenomic associations.^1^, ^7^ Both whole transcriptome sequencing data and postcontrast T1‐weighted MR images from the Cancer Genome Atlas (TCGA) were analyzed to explore differentially expressed genes and determine a CE‐related signature. Data from the Chinese Glioma Genome Atlas (CGGA) were utilized to validate the derived signature diagnostically and prognostically. The prognostic value of quantitative radiomic features of CE was also preliminarily investigated in this study.

## 2 MATERIALS AND METHODS

### 2.1 Patients and samples

Ninety‐one patients (49 men; median age, 45 years; range, 22‐75 years; and 42 women, median age, 50 years; range, 22‐74 years) diagnosed with AG were extracted from TCGA database ( http://cancergenome.nih.gov) and comprised the training set (Figure S1). Additionally, clinical characteristics of 64 cases (40 men; median age, 42 years; range, 20‐70 years; and 24 women, median age, 44.5 years; range, 18‐67 years) diagnosed with AG were obtained from the CGGA database ( http://www.cgga.org.cn) and were deemed the validation set. Only those cases with both RNA‐sequencing data and MR imaging data were included in this retrospective study. The study was approved by our institutional review board.

### 2.2 Image acquisition and evaluation

**Figure 1:**
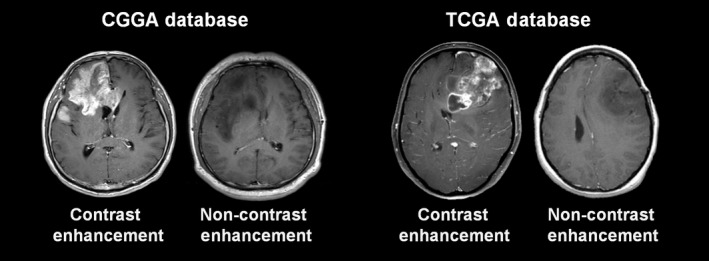
Examples of Contrast Enhancement and Noncontrast Enhancement Images for Analyses. CGGA, Chinese Glioma Genome Atlas; TCGA, The Cancer Genome Atlas

The Cancer Genome Atlas MR images of AGs were downloaded from the Cancer Imaging Archive (TCIA, http://www.cancerimagingarchive.net). CGGA MR images of AGs were obtained from the CGGA imaging database ( http://www.cgga.org.cn) administered by the Chinese Glioma Cooperation Group. MR images in CGGA patients were generally obtained with a Trio 3.0T scanner (Siemens, Erlangen, Germany). Tumor CE was defined as newly emerged, unequivocally increased signal intensity on the T1‐weighted image following intravenous contrast administration when compared to noncontrast T1 images. Nonenhancement (nCE) was defined as no apparent enhancement in tumors on postcontrast T1‐weighted images, compared with regular T1‐weighted images (Figure 1). The presentation of tumor CE was evaluated by two experienced neuroradiologists (X.C. and J.M., both with more than 15 years of neuroradiological experiences) blinded to the patients‧ clinical information. A third senior neuroradiologist (S.L. with more than 20 years of neuroradiological experiences) arbitrated when necessary.

### 2.3 RNA sequencing and molecular analyses

Chinese Glioma Genome Atlas RNA sequencing was performed as previously described.^8^ Briefly, libraries were sequenced on the Illumina HiSeq 2000 platform using the 101‐bp pair‐end sequencing strategy. Short sequence reads were aligned to the human reference genome (Hg19Refseq) using the Burrows‐Wheeler Aligner (BWA, Version 0.6.2‐r126).^9^ *IDH* mutations and O‐6‐methylguanine‐DNA methyltransferase promoter methylation were assessed by pyrosequencing.^10^ TCGA RNA sequencing data and corresponding molecular profiles^11^ were obtained from TCGA database. The genes available for our genetic analysis were more than 20 000 both in the CGGA and TCGA databases.

### 2.4 Image‐genomic analysis

RNA sequencing data of CE and nCE patients were subjected to gene set enrichment analysis (GSEA). GSEA was performed using dedicated software ( http://www.broadinstitute.org/gsea). Annotated gene sets were downloaded from the Molecular Signatures Database v5.1 (MSigDB) ( http://www.broad.mit.edu/gsea/msigdb/). Differentially expressed genes were selected by significance analysis of microarray (SAM) conducted with the R programming language ( http://cran.r-project.org), with a false discovery rate (FDR) <0.05. Heat maps of differential genes were constructed using Gene Cluster^12^ and Gene Tree View software.^13^ Kaplan‐Meier survival analysis was applied to estimate the survival distributions. Gene Ontology (GO) analysis was performed using the online Database for Annotation, Visualization, and Integrated Discovery (DAVID, http://david.ncifcrf.gov/).^14^ GO terms were visualized by EnrichmentMap^15^ and AutoAnnotate^16^ plugins in Cytoscape software.^17^

### 2.5 Signature development and validation

Generalized Linear Models (GLM) (*Y* = expr_gene1_ × β_gene1_ +expr_gene2_ × β_gene2_ + … + expr _gene n_ × β_gene n_ + ε) was calculated using Matlab (2014a) software (MathWorks, Natick, MA, USA). For each patient, “Y” represents MR manifestation (1 indicates CE tumors, 0 indicates nCE tumors), while “expr” represents the expression level for each candidate gene. β represents the model parameter to be estimated, and ε is the estimated residual.

The GLM algorithm was repeatedly applied to extract a gene signature containing those genes that best predicted CE in tumors. Using the dimensionality reduction principle, the gene with the highest *P* value when classifying CE and nCE AG was eliminated from the model each time, until a target number of genes were left. A series of receiver operating characteristic curves were delineated based on the screened genes. Associated genes with the maximal area under the curve (AUC) were established as the CE specific signature. The signature derived from the training set was subsequently applied to the CGGA for validation.

The prognostic values of candidate genes in patients with AG were calculated by least absolute shrinkage and selection operator (LASSO) algorithm. For preliminary analysis, patients with overall survival times less than 30 days were excluded. The selected genes were used in developing a linear combination weighted by their respective coefficients generated by Lasso‐Cox model.^18^ The risk score for overall survival time of each individual was calculated as follows:

$$\begin{array}{cc} \text{Risk}\;\;=\; & {\text{expr}}_{\mathrm{gene}1}\times {\text{coefficient}}_{\mathrm{gene}1}+\;{\text{expr}}_{\mathrm{gene}2}\\ & \times \;{\text{coefficient}}_{\mathrm{gene}2}+\ldots +\;{\text{expr}}_{\mathrm{gene}7}\times \;{\text{coefficient}}_{\mathrm{gene}7}. \end{array}$$

We next divided patients in the training dataset into high‐risk and low‐risk groups using the median mRNA signature risk score as the cutoff point; patients with higher risk scores were posited to have poor survival. The same coefficients and median risk score cutoff was applied to the validation cohort.

### 2.6 Radiogenomic analysis of quantitative radiomic features of CE

In TCGA database, the CE mask was manually delineated by two experienced neuroradiologists on CE images using MRIcro software ( http://www.mccauslandcenter.sc.edu/crnl/mricro). The Dice coefficient was used to measure the discrepancy between tumor masks, and a senior neuroradiologist made a final decision about the tumor border when the discrepancy was >5%.^19^ Fifty‐five quantitative radiomic features were extracted from the CE mask using the method as previously described.^20^ The features can be classified into three groups: (a) first‐order statistics, which quantitatively measure the distribution of voxel intensities within the image; (b) shape‐ and size‐based features, which reflect the shape and size of the tumor region; and (c) textual features, which can quantify intratumor heterogeneity differences.

Firstly, univariate Cox regression was performed on the radiomic features individually in order to screen prognostic features. Subsequently, Pearson correlation algorithm was used to screen genes that were associated with the selected radiomic features. The top 500 positive/negative genes that were significantly associated with each feature were subjected to gene ontology analysis to reveal the underlying biological processes involved in each feature. When the hazard ratio of a prognostic feature was larger than 1, gene ontology analysis was performed on the positive associated genes. When the hazard ratio was less than 1, gene ontology analysis was performed on the negative associated genes. Correlations between the selected prognostic features were calculated using Spearman's correlation analysis. *P* < 0.05 was considered statistically significant.

## 3 RESULTS

### 3.1 Clinical and radiological characteristics

Clinical and radiological features of all 91 TCGA patients (training set) and 64 CGGA patients (validation set) are summarized in Table 1. No significant difference was observed between the CE and nCE groups with respect to age, sex, and O‐6‐methylguanine‐DNA methyltransferase (MGMT) promoter methylation status in both the training and validation sets (*P *>* *0.05). Mutations in IDH,^21^ a crucial prognostic biomarker for gliomas, were more common in the nCE group in both the training (*P *=* *0.038) and validation (*P *=* *0.043) sets. Additionally, there was no difference about 1p/19q status between the CE and nCE groups in the training set (*P *>* *0.05), while the frequency of 1p/19q co‐deletion was higher in the nCE group in the validation set (*P *=* *0.005). CE patients in the CGGA dataset consistently presented high rates of *IDH* mutation (*P *=* *0.043) and undifferentiated *MGMT* promoter methylation (*P *=* *0.451). The frequency of chromosome 1p/19q co‐deletion was higher in CGGA nCE patients (*P *=* *0.005).

**Table 1:** Clinical characteristics of anaplastic gliomas in TCGA and CGGA patients

	TCGA database			CGGA database		
	CE (n = 58)	nCE (n = 33)	*P‐*value^a^	CE (n = 47)	nCE (n = 17)	*P*‐value^a^
Age (years)						
≥50	27	12	0.384	17	3	0.226
<50	31	21		30	14	
Sex						
Male	33	16	0.514	29	11	>0.999
Female	25	17		18	6	
WHO classification						
A						
A, *IDH* ^MUT^	8	14		3	2	
A, *IDH* ^WT^	15	5		10	3	
A, *NOS*	0	0		2	0	
O						
O	17	5		3	6	
O, *NOS*	6	3		1	1	
OA, *NOS*	12	6		28	5	
Location						
Left	26	14	0.501	21	7	0.548
Right	29	19		24	8	
Midline	2	0		2	2	
NA	1	0		0	0	
*IDH* status						
Mutant	35	27	**0.038**	17	12	**0.043**
Wild type	23	6		27	5	
NA	0	0		3	0	
1p/19q status						
Codeletion	17	6	0.318	4	7	**0.005**
Non‐codeletion	41	27		43	10	
MGMT promoter						
Methylation	41	28	0.202	23	11	0.451
Wild type	17	5		19	4	
NA	0	0		5	2	

A, astrocytoma; CE, contrast enhancement; CGGA, Chinese Glioma Genome Atlas; MGMT, O^6^‐Methylguanine‐DNA methyltransferase; NA, data not available; NOS, not other specified; O, oligodendroglioma; OA, oligodendroglioma; TCGA, The Cancer Genome Atlas.

a Result of Fisher's exact or chi‐square test.

The bold values indicate that they are statistically significant (< 0.05).

### 3.2 GSEA‐identified gene functions associated with tumor enhancement

**Figure 2:**
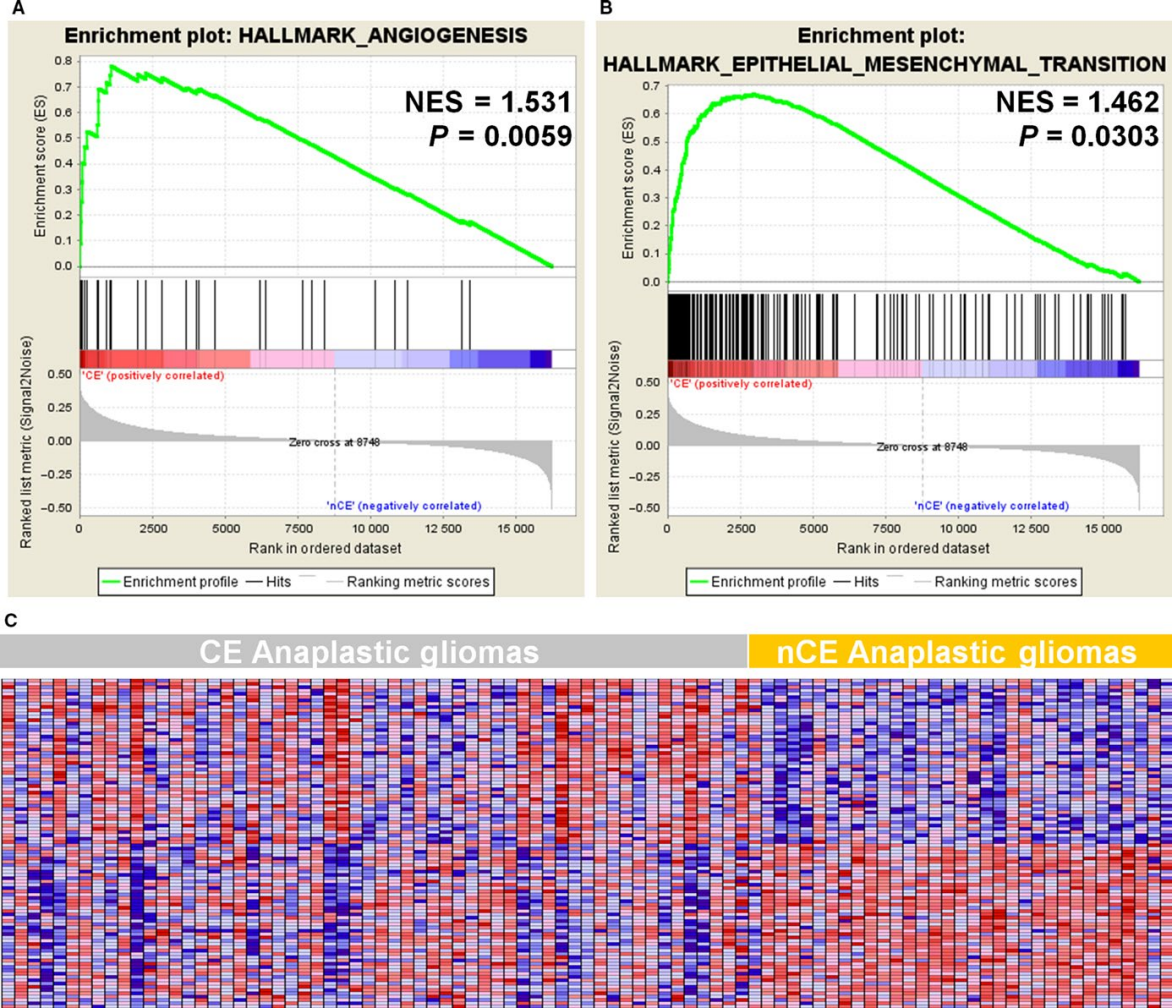
Hallmark Gene Sets Differentially Enriched in the Contract Enhancement (CE) and Non‐CE (nCE) Subgroups, Analyzed by Gene Set Enrichment Analysis of the Cancer Genome Atlas RNA‐Sequencing Data. NES, normalized enrichment score

To characterize CE properties, we divided TCGA AG patients into CE and nCE groups according to the postcontrast T1‐weighted MR images presentation. Hallmark gene sets representing specific well‐defined biological states were acquired from the MSigDB and analyzed by GSEA. Results suggested that CE patients had upregulated angiogenesis (normalized enrichment score = 1.531, *P *=* *0.0059) and epithelial‐mesenchymal transition (normalized enrichment score = 1.462, *P *=* *0.0303) (Figure 2). Other gene set enrichment analyses were also performed. Significant enrichment was observed in genes associated with immune response, G1/S transition of the mitotic cell cycle, extracellular matrix structural components, and focal adhesion; all are key biological processes, molecular events, and pathways involved in tumor malignancy (Table S1). The canonical pathways seen in the CE vs nCE differentially expressed genes include lymph‐angiogenesis pathway, extracellular matrix organization, EPHA2 FWD pathway, focal adhesion, etc.

### 3.3 Screening and annotation of differential genes

**Figure 3:**
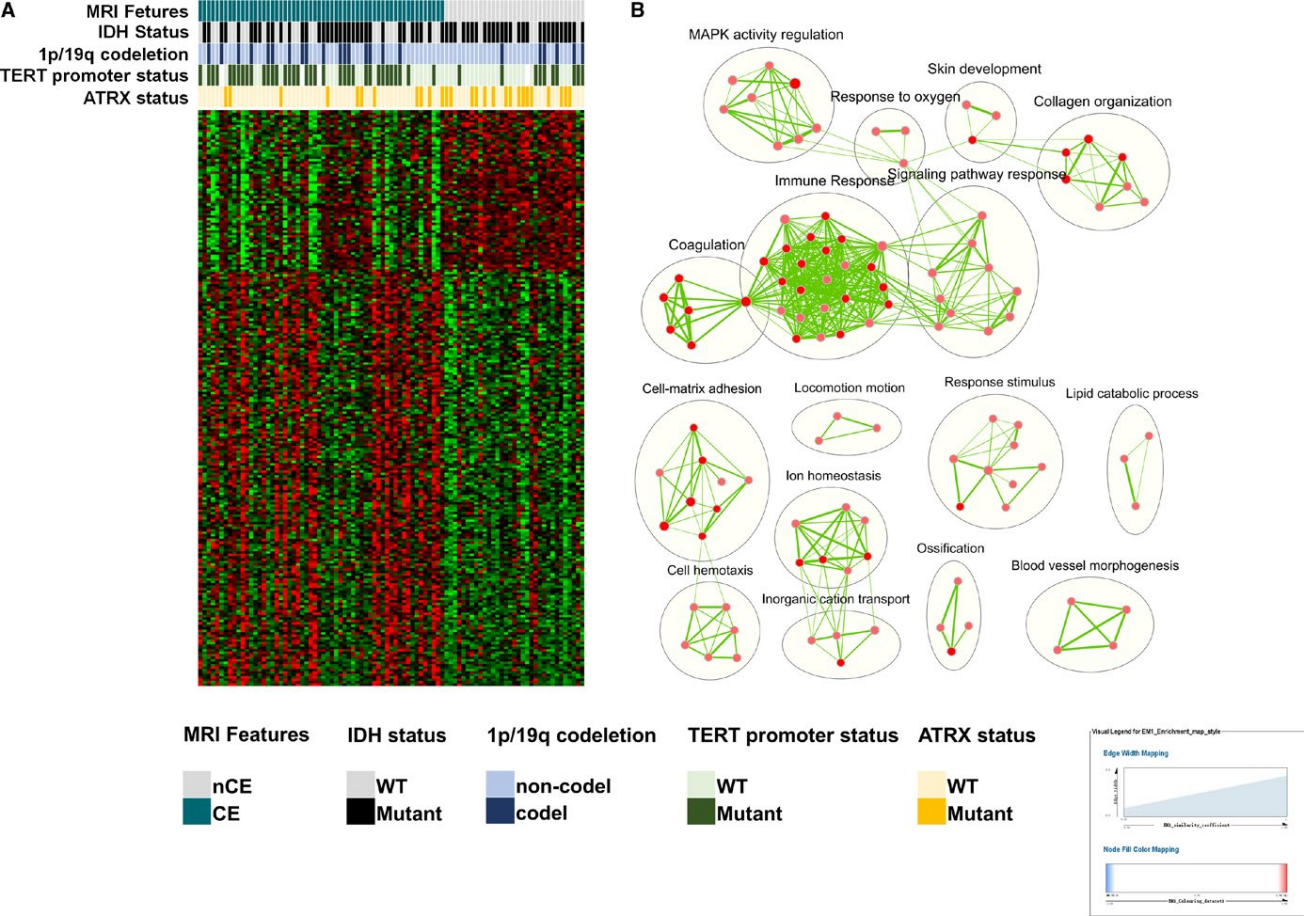
Differential Genes Screening and Gene Ontology. A, Heat map of 237 differential expressed genes and corresponding molecular‐pathological biomarkers. CE, contrast enhancement; CGGA, Chinese Glioma Genome Atlas; nCE, noncontrast enhancement; WT, wild type; TCGA, The Cancer Genome Atlas. B, Visualized gene ontology terms of differential genes of biological processes between the two subgroups. These biological processes, including immune response, adhesion, locomotion, and blood vessel morphogenesis, which is consistent with the consequence of gene set enrichment analysis

To further investigate CE‐associated molecular alterations, we utilized the SAM method for differentially expressed genes filtering. After excluding genes with FDR ≥ 0.05 and fold change <20%, 169 and 68 genes positively and negatively correlating with CE, respectively, were selected. Intriguingly, in the genes that are positively corelated with CE, these are a series of well‐documented genes that encode proteins promoting glioma cell malignancy, such as MMP9, an enzyme involved in the breakdown of the extracellular matrix; LIF, a cytokine that affects cell growth; and TWIST1, a transcription factor involved in epithelial‐mesenchymal transition. A heat map of these 237 genes was constructed (Figure 3A). Next, 169 CE‐positive genes were subjected to DAVID analysis (Table S2). Visualized GO terms of biological processes with *P *<* *0.05 were established (Figure 3B). Consistently, enriched CE‐associated genes were mainly those involved in immune response, vascular development, and cell adhesion.

### 3.4 Signature associated with CE

**Figure 4:**
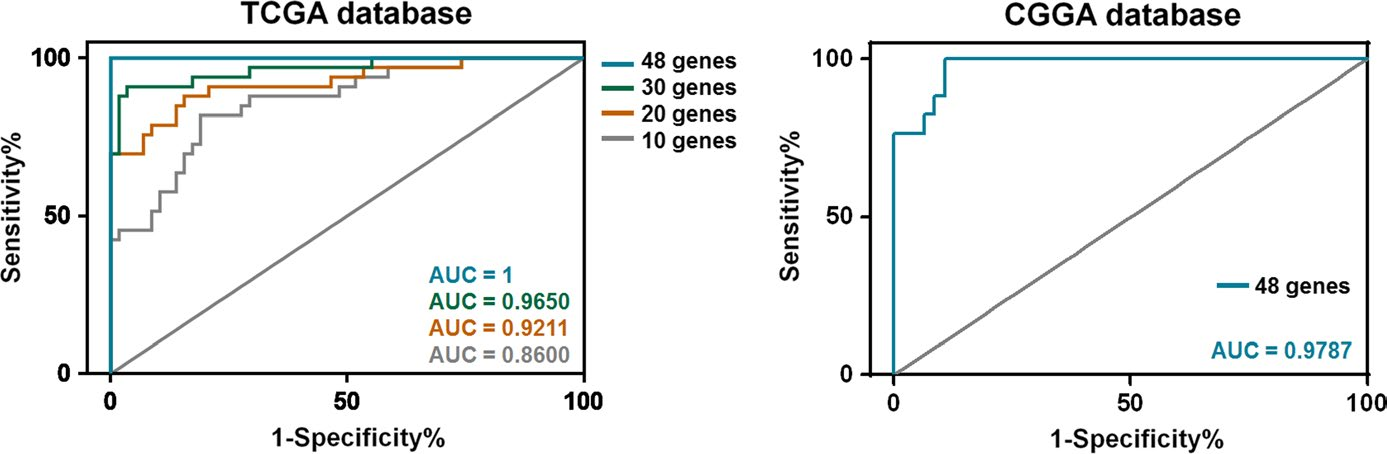
Contrast Enhancement (CE)‐Related Signature Establishment and Validation. A series of receiver operating characteristic curves were delineated based on the retrieved genes. The areas under the curve (AUC) for 10 genes, 20 genes, 30 genes, and 48 genes were 0.86, 0.92, 0.97, and 1.00, respectively. The predictive capability of the established signature (45 genes, excluding *DNAH11*,*LOC283314*, and *LOC285370*) was validated using the Chinese Glioma Genome Atlas RNA‐sequencing data. The AUC for this CE‐related signature was 0.9787

Generalized Linear Model algorithms were performed to extract a meaningful gene signature from 237 genes associated with CE tumors. A group of 48 genes was selected (Table S3). Among them, 42 were CE‐positive related; these included *POSTN*,* ESM1*,* KMO*, and other previously reported oncogenes. For validation of this CE‐related signature, we applied these genes to the CGGA dataset using the GLM model. Because of the discrepancy in sequencing, three genes (*DNAH11*,* LOC283314*, and *LOC285370*) were not found in the CGGA dataset. The AUC for the remaining 45 genes in terms of classifying CGGA CE‐ and nCE‐AG patients was 0.9787; this affirmed the CE specificity of this signature (Figure 4).

### 3.5 Prognostic role of the radiogenomic signature

**Figure 5:**
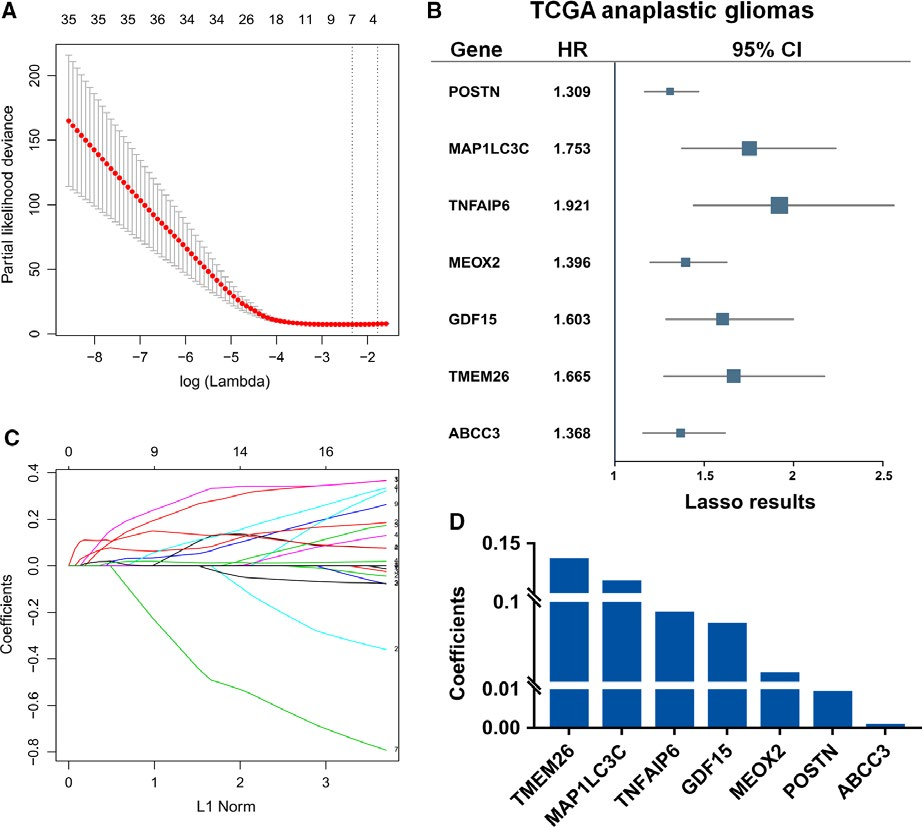
Construction of Contrast Enhancement‐Based Prognostic Gene Set. A, The 10‐folds cross‐validation for LASSO‐Cox analysis identified seven genes signature. B, The seven genes were also significant in univariate Cox regression analysis. C‐D, The coefficients for the significant genes derived from LASSO‐Cox model

To further explore the prognostic effect of CE‐related genes, we extracted a compact signature consisting of genes using LASSO‐Cox regression analysis with 10‐fold cross‐validation (Figure 5A). Seven genes with a nonzero coefficient were TMEM26 (0.1332), MAP1LC3C (0.1112), TNFAIP6 (0.0872), GDF15 (0.0746), MEOX2 (0.0188), POSTN (0.0090), and ABCC3 (0.0006) (Figure 5, Table S4). Therefore, the risk score could be calculated using the following formula: risk score = expr _TMEM26_ × 0.1332 + expr _MAP1LC3C_ × 0.1112 + expr _TNFAIP6_ × 0.0872 + expr _GDF15_ × 0.0746 + expr _MEOX2_ × 0.0188 + expr _POSTN_ × 0.0090 + expr _ABCC3_ × 0.0006.

**Figure 6:**
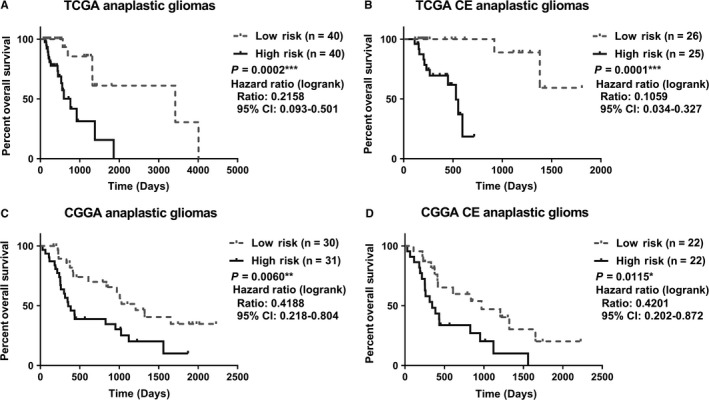
Prognostic Implication of the Gene Signature in the Training and Validation Sets. Patients were divided into high‐risk and low‐risk groups. Anaplastic gliomas (AG) patients with high‐risk scores had worse prognoses than low‐risk patients in both TCGA and CGGA datasets. Moreover, postcontrast enhanced AG patients were stratifiable by this risk signature. CE, contrast enhancement; CGGA, Chinese Glioma Genome Atlas; HR, hazard ratio (95% confidence interval); TCGA, The Cancer Genome Atlas

Next, we categorized patients into high‐risk and low‐risk groups according risk score; the median risk score was the cutoff. Intriguingly, in the TCGA cohort, the overall survival curves of the high‐ and low‐risk groups were significantly separated (Figure 6A, *P* = 0.0002). Moreover, CE patients with high‐risk scores had worse overall survival rates than low‐risk CE patients in the TCGA cohort (Figure 6B, *P* = 0.0001), which further emphasized the prognostic value of the CE‐related gene expression signature. Consistently, the overall survival of the high‐risk group was markedly poorer than that of the low‐risk group both in AG patents and enhanced CE patients, respectively, in the CGGA cohort (Figure 6C,D, *P* = 0.0060 and *P* = 0.0115).

### 3.6 Radiogenomic analysis of quantitative radiomic features of CE

Using the univariate Cox regression, nine prognostic radiomic features were identified (Table S5). Intriguingly, all of the prognostic features were textual features (Energy, Entropy, High Gray Level Run Emphasis, Informational Measure of Correlation 1, Long Run High Gray Level Emphasis, Low Gray Level Run Emphasis, Maximum Probability, Short Run Low Gray Level Emphasis, Sum Entropy).

Figure S2 shows that most of the prognostic features were associated with biological processes such as angiogenesis, cell proliferation, cell migration, response to hypoxia, etc. Correlation analysis revealed that many significant correlations existed between these features (Figure S3), which could explain why their associated biological processes were so similar.

## 4 DISCUSSION

Postcontrast T1‐weighted MR imaging is an optimal radiological modality for diagnosis and clinical management of malignant gliomas. The leakage of the contrast agent Gd‐DTPA, which is attributed to the infiltration of tumor cells and focal abnormalities in the blood‐brain barrier, produces an increasing signal on T1‐weighted imaging. Previous studies suggested that increased neovascular permeability may also contribute to post‐CE.^22^ Furthermore, an early radiogenomic study revealed that contrast‐enhanced tumor volume was strongly associated with poor survival in glioblastoma.^23^ Hence, CE could serve as a noninvasive indicator of a tumor's biological process. However, the potential genetic alterations and corresponding molecular pathways of contrast enhanced AG remain unclear. A previous study revealed that the presence of CE was associated with *IDH* mutation in glioblastomas.^24^ In another study, it was found that CE could be associated with several proangiogenic and edema‐related genes, including neuronal pentraxin‐2 and vascular endothelial growth factor in GBM patients.^25^ These studies increased the impetus for an integrative analysis of radiological presentation and multi‐omics data. In the present study, we comprehensively combined classical molecular‐pathological biomarkers, whole genome transcriptome sequencing, clinical characteristics, radiological manifestations, and radiomics for the first time, and established a CE‐related gene expression signature that could predict malignant behaviors and unsatisfactory prognoses.

The genes that are differentially expressed in CE compared to nCE tumors have specific biological functions. Several genes have clear associations with tumorigenesis in glioma or other types of carcinoma. *POSTN*, encoding secreted matricellular protein Periostin, is critical for epithelial‐mesenchymal transition, tumor angiogenesis, and metastasis.^26^ A pioneer radiogenomic study found that POSTN was the top upregulated gene that could reflect edema/cellular invasion, and revealed that high expression of POSTN resulted in poor overall survival and progression‐free survival in GBM patients.^27^ MZ‐1, a neutralizing monoclonal antibody to POSTN, showed significant growth inhibition both in vivo and in vitro,^28^ thereby providing an alternative approach in clinical management of CE patients. *KMO* is a pivotal enzyme in the kynurenine‐mediated tryptophan degradation pathway; it positively regulates proliferation, migration, and invasion of tumor cells, and may serve as a novel prognostic marker in various cancers.^29^, ^30^ Recently, investigators revealed the crystal structure of Saccharomyces cerevisiae KMO, in the free form and in complex with the tight‐binding inhibitor UPF 648,^31^ which will promote the search for new KMO inhibitors in targeted therapies against neurodegenerative diseases and tumor.

Two sets of hallmark genes were meaningfully enriched when comparing CE to nCE subgroups using GSEA analysis. Epithelial‐mesenchymal transition plays a prominent role in epithelial cell invasion, resistance to apoptosis, degradation of the limiting basement membrane, and tumor dissemination.^32^, ^33^ Through this process, glioma cells can achieve augmented invasion and increased blood‐brain barrier damage, leading to leakage of contrast agents. Another hallmark gene set generated by GSEA analysis concerned angiogenesis. Typically, blood vessels formed owing to an unbalanced mix of proangiogenic signals are misshapen, as evidenced by precocious capillary sprouting, convoluted and excessive vessel branching, distorted and enlarged vessels, erratic blood flow, and abnormal levels of endothelial cell proliferation and apoptosis.^34^, ^35^ Therefore, these newly formed vessels can leak and cause the accumulation of radiocontrast agent in surrounding tissues, shortening the longitudinal relaxation time of neighboring water protons. Hence, therapeutically targeting these angiogenic factors may provide an effective approach for CE glioma management. GSEA results also suggested that the G1/S transition phase of the mitotic cell cycle, positive regulation of cell proliferation, and other tumor‐promoting processes may contribute to postcontrast enhancement; GO analysis demonstrated consistency with the GSEA. Notably, the immune response appeared to be involved in postcontrast enhancement. Immunity‐associated genes, including chemokine and the chemokine receptors *CCR2*,^36^, ^37^, ^38^ *CCR7*,^39^ and *CXCL9* 40 are well‐documented oncogenes in numerous cancers and are involved in mediating the crosstalk between tumors and their microenvironment, as well as in promoting metastasis.

A 48‐gene signature, based on 237 differential genes, was established using the GLM algorithm. These compact genes were found to be associated with inflammatory response, cell adhesion, microtubule motor activity, angiogenesis, positive regulation of cell proliferation, and positive regulation of cell division; this was consistent with the GSEA and GO results. Furthermore, WNT signaling associated genes, such as *HIST1H4J*,* WNT16*, and *WNT7B* were significantly involved, suggesting a role for the WNT signaling pathway in promoting postcontrast enhancement. Notably, the derived prognostic signature could significantly stratify enhanced vs nonenhanced AG patients post contrast administration. This finding promotes the gene signature as a convenient prognostic tool for neurological clinicians.

With high‐throughput computing, it is now possible to rapidly extract many quantitative features from medical images (known as radiomics), providing a powerful tool of associating images with underlying biological processes or clinical events.^41^ In the present study, the prognostic value of the quantitative radiomic features of CE was also investigated. Nine prognostic features were identified using the univariate Cox regression model, and all of the nine features were textural features (group 3). We hypothesize that textural features are more capable of capturing the prognostic information in patients with CE AG, since textural features have exhibited powerful prognostic value in many other studies.^20^, ^42^, ^43^ Further radiogenommic analysis revealed that all the nine prognostic features were associated with angiogenesis, which indicates that angiogenesis might be a suitable therapeutic target for patients with CE AG.

Several limitations should be noted in the present study. First, the associations and mechanistic roles of the candidate genes have not all been experimentally confirmed in previous studies. Future larger‐scale studies with mechanistic exploration are required to correlate observed imaging features with biological function. As for the prognostic signature, classifying patients using their prognosis may develop a more powerful signature, and we will try this method in our future studies. Finally, the findings of radiogenomic analysis was preliminary. More quantitative radiomic features and more MR sequences should be involved in the future.

In summary, this study emphasized the relevance of whole genome transcriptomes to radiological manifestation. CE, one of the most valuable radiological features of malignant gliomas, was positively associated with tumor angiogenesis and epithelial‐mesenchymal transition. We identified 48 genes derived from a pool of 237 differentially expressed genes that may serve as a CE‐specific signature. Meanwhile, we also showed that a simplified signature consisting of seven genes can be used to reliably classify AG patients according to prognosis. Finally, we investigated the prognostic radiomic features of CE and revealed the underlying biological processes of the features. Therefore, our results illustrate an intrinsic correlation between radiological, molecular, and phenotypic observations, and highlight the value of the radiogenomic approach to prognostication and customized treatment guidance.

## CONFLICT OF INTEREST

There were no conflicts of interest to disclose.

## References

[1] Reyes‐Botero G , Dehais C , Idbaih A , et al. Contrast enhancement in 1p/19q‐codeleted anaplastic oligodendrogliomas is associated with 9p loss, genomic instability, and angiogenic gene expression. Neuro Oncol. 2014;16(5):662‐670. 24353325 10.1093/neuonc/not235 PMC3984545

[2] Naeini KM , Pope WB , Cloughesy TF , et al. Identifying the mesenchymal molecular subtype of glioblastoma using quantitative volumetric analysis of anatomic magnetic resonance images. Neuro Oncol. 2013;15(5):626‐634. 23444259 10.1093/neuonc/not008 PMC3635524

[3] Beiko J , Suki D , Hess KR , et al. IDH1 mutant malignant astrocytomas are more amenable to surgical resection and have a survival benefit associated with maximal surgical resection. Neuro Oncol. 2014;16(1):81‐91. 24305719 10.1093/neuonc/not159 PMC3870823

[4] Jiang T , Mao Y , Ma W , et al. CGCG clinical practice guidelines for the management of adult diffuse gliomas. Cancer Lett. 2016;375(2):263‐273. 26966000 10.1016/j.canlet.2016.01.024

[5] Woodard GA , Ray KM , Joe BN , Price ER . Qualitative Radiogenomics: Association between Oncotype DX Test Recurrence Score and BI‐RADS Mammographic and Breast MR Imaging Features. Radiology. 2018;286(1):60‐70. 28885890 10.1148/radiol.2017162333

[6] Hu LS , Ning S , Eschbacher JM , et al. Radiogenomics to characterize regional genetic heterogeneity in glioblastoma. Neuro Oncol. 2017;19(1):128‐137. 27502248 10.1093/neuonc/now135 PMC5193022

[7] Chaichana KL , Kosztowski T , Niranjan A , et al. Prognostic significance of contrast‐enhancing anaplastic astrocytomas in adults. J Neurosurg. 2010;113(2):286‐292. 20302391 10.3171/2010.2.JNS091010

[8] Bao ZS , Chen HM , Yang MY , et al. RNA‐seq of 272 gliomas revealed a novel, recurrent PTPRZ1‐MET fusion transcript in secondary glioblastomas. Genome Res. 2014;24(11):1765‐1773. 25135958 10.1101/gr.165126.113 PMC4216918

[9] Li H , Durbin R . Fast and accurate long‐read alignment with Burrows‐Wheeler transform. Bioinformatics. 2010;26(5):589‐95. 20080505 10.1093/bioinformatics/btp698 PMC2828108

[10] Zhang CB , Bao ZS , Wang HJ , et al. Correlation of IDH1/2 mutation with clinicopathologic factors and prognosis in anaplastic gliomas: a report of 203 patients from China. J Cancer Res Clin Oncol. 2014;140(1):45‐51. 24149775 10.1007/s00432-013-1519-9 PMC11824052

[11] Ceccarelli M , Barthel FP , Malta TM , et al. Molecular profiling reveals biologically discrete subsets and pathways of progression in diffuse glioma. Cell. 2016;164(3):550‐563. 26824661 10.1016/j.cell.2015.12.028 PMC4754110

[12] de Hoon MJ , Imoto S , Nolan J , Miyano S . Open source clustering software. Bioinformatics. 2004;20(9):1453‐1454. 14871861 10.1093/bioinformatics/bth078

[13] AJ . Java Treeview‐‐extensible visualization of microarray data. Bioinformatics. 2004;20(17):3246‐3248. 15180930 10.1093/bioinformatics/bth349

[14] Huang dW , Sherman BT , Lempicki RA . Systematic and integrative analysis of large gene lists using DAVID bioinformatics resources. Nat Protoc. 2009;4(1):44‐57. 19131956 10.1038/nprot.2008.211

[15] Isserlin R , Merico D , Voisin V , Bader GD . Enrichment Map—a Cytoscape app to visualize and explore OMICs pathway enrichment results. F1000Res. 2014; 3: 141. 25075306 10.12688/f1000research.4536.1 PMC4103489

[16] Kucera M , Isserlin R , Arkhangorodsky A , Bader GD . AutoAnnotate: a Cytoscape app for summarizing networks with semantic annotations. F1000Res. 2016;5:1717. 27830058 10.12688/f1000research.9090.1 PMC5082607

[17] Shannon P , Markiel A , Ozier O , et al. Cytoscape: a software environment for integrated models of biomolecular interaction networks. Genome Res. 2003;13(11):2498‐2504. 14597658 10.1101/gr.1239303 PMC403769

[18] Huang Y , Liu Z , He L , et al. Radiomics signature: a potential biomarker for the prediction of disease‐free survival in early‐stage (I or II) non‐small cell lung cancer. Radiology. 2016;281:947‐957. 27347764 10.1148/radiol.2016152234

[19] Wang Y , Qian T , You G , et al. Localizing seizure‐susceptible brain regions associated with low‐grade gliomas using voxel‐based lesion‐symptom mapping. Neuro Oncol. 2015;17(2):282‐288. 25031032 10.1093/neuonc/nou130 PMC4288515

[20] Aerts HJ , Velazquez ER , Leijenaar RT , et al. Decoding tumour phenotype by noninvasive imaging using a quantitative radiomics approach. Nat Commun. 2014;5:4006. 24892406 10.1038/ncomms5006 PMC4059926

[21] Yang P , Cai J , Yan W , et al. Classification based on mutations of TERT promoter and IDH characterizes subtypes in grade II/III gliomas. Neuro Oncol. 2016;18:1099‐1108. 26957363 10.1093/neuonc/now021 PMC4933482

[22] Matar E , Cook RJ , Fowler AR , et al. Post‐contrast enhancement as a clinical indicator of prognosis in patients with anaplastic astrocytoma. J Clin Neurosci. 2010;17(8):993‐996. 20605464 10.1016/j.jocn.2009.12.015

[23] Gutman DA , Cooper LA , Hwang SN , et al. MR imaging predictors of molecular profile and survival: multi‐institutional study of the TCGA glioblastoma data set. Radiology. 2013;267(2):560‐569. 23392431 10.1148/radiol.13120118 PMC3632807

[24] Wang K , Wang Y , Fan X , et al. Radiological features combined with IDH1 status for predicting the survival outcome of glioblastoma patients. Neuro Oncol. 2016;18(4):589‐597. 26409566 10.1093/neuonc/nov239 PMC4799681

[25] Pope WB , Chen JH , Dong J , et al. Relationship between gene expression and enhancement in glioblastoma multiforme: exploratory DNA microarray analysis. Radiology. 2008;249(1):268‐277. 18796682 10.1148/radiol.2491072000 PMC2798090

[26] Zhou W , Ke SQ , Huang Z , et al. Periostin secreted by glioblastoma stem cells recruits M2 tumour‐associated macrophages and promotes malignant growth. Nat Cell Biol. 2015;17(2):170‐182. 25580734 10.1038/ncb3090 PMC4312504

[27] Zinn PO , Mahajan B , Majadan B , et al. Radiogenomic mapping of edema/cellular invasion MRI‐phenotypes in glioblastoma multiforme. PLoS ONE. 2011;6(10):e25451. 21998659 10.1371/journal.pone.0025451 PMC3187774

[28] Zhu M , Saxton RE , Ramos L , et al. Neutralizing monoclonal antibody to periostin inhibits ovarian tumor growth and metastasis. Mol Cancer Ther. 2011;10(8):1500‐1508. 21670235 10.1158/1535-7163.MCT-11-0046

[29] Jin H , Zhang Y , You H , et al. Prognostic significance of kynurenine 3‐monooxygenase and effects on proliferation, migration, and invasion of human hepatocellular carcinoma. Sci Rep. 2015;5:10466. 26099564 10.1038/srep10466 PMC4479133

[30] Adams S , Teo C , McDonald KL , et al. Involvement of the kynurenine pathway in human glioma pathophysiology. PLoS ONE. 2014;9(11):e112945. 25415278 10.1371/journal.pone.0112945 PMC4240539

[31] Amaral M , Levy C , Heyes DJ , et al. Structural basis of kynurenine 3‐monooxygenase inhibition. Nature. 2013;496(7445):382‐385. 23575632 10.1038/nature12039 PMC3736096

[32] Polyak K , Weinberg RA . Transitions between epithelial and mesenchymal states: acquisition of malignant and stem cell traits. Nat Rev Cancer. 2009;9(4):265‐273. 19262571 10.1038/nrc2620

[33] Kahlert UD , Nikkhah G , Maciaczyk J . Epithelial‐to‐mesenchymal(‐like) transition as a relevant molecular event in malignant gliomas. Cancer Lett. 2013;331(2):131‐138. 23268331 10.1016/j.canlet.2012.12.010

[34] Hanahan D , Weinberg RA . Hallmarks of cancer: the next generation. Cell. 2011;144(5):646‐674. 21376230 10.1016/j.cell.2011.02.013

[35] Nagy JA , Chang SH , Shih SC , Dvorak AM , Dvorak HF . Heterogeneity of the tumor vasculature. Semin Thromb Hemost. 2010;36(3):321‐331. 20490982 10.1055/s-0030-1253454 PMC3278036

[36] Rao Q , Chen Y , Yeh CR , et al. Recruited mast cells in the tumor microenvironment enhance bladder cancer metastasis via modulation of ERβ/CCL2/CCR2 EMT/MMP9 signals. Oncotarget. 2016;7(7):7842‐7855. 26556868 10.18632/oncotarget.5467 PMC4884958

[37] Li X , Yao W , Yuan Y , et al. Targeting of tumour‐infiltrating macrophages via CCL2/CCR2 signalling as a therapeutic strategy against hepatocellular carcinoma. Gut. 2015;66:157‐167. 26452628 10.1136/gutjnl-2015-310514

[38] Nywening TM , Wang‐Gillam A , Sanford DE , et al. Targeting tumour‐associated macrophages with CCR2 inhibition in combination with FOLFIRINOX in patients with borderline resectable and locally advanced pancreatic cancer: a single‐centre, open‐label, dose‐finding, non‐randomised, phase 1b trial. Lancet Oncol. 2016;17:651‐662. 27055731 10.1016/S1470-2045(16)00078-4 PMC5407285

[39] Shields JD , Kourtis IC , Tomei AA , Roberts JM , Swartz MA . Induction of lymphoidlike stroma and immune escape by tumors that express the chemokine CCL21. Science. 2010;328(5979):749‐752. 20339029 10.1126/science.1185837

[40] Ding Q , Xia Y , Ding S , Lu P , Sun L , Liu M . An alternatively spliced variant of CXCR3 mediates the metastasis of CD133^+^ liver cancer cells induced by CXCL9. Oncotarget. 2016;7(12):14405‐14414. 26883105 10.18632/oncotarget.7360 PMC4924724

[41] Gillies RJ , Kinahan PE , Hricak H . Radiomics: images are more than pictures. They are data. Radiology. 2016;278(2):563‐577. 26579733 10.1148/radiol.2015151169 PMC4734157

[42] Kickingereder P , Burth S , Wick A , et al. Radiomic profiling of glioblastoma: identifying an imaging predictor of patient survival with improved performance over established clinical and radiologic risk models. Radiology. 2016;280(3):880‐889. 27326665 10.1148/radiol.2016160845

[43] Zhou H , Vallières M , Bai HX , et al. MRI features predict survival and molecular markers in diffuse lower‐grade gliomas. Neuro Oncol. 2017;19(6):862‐870. 28339588 10.1093/neuonc/now256 PMC5464433

