# Supplementary figures and images for: Molecular profiles of tumor contrast enhancement: A radiogenomic analysis in anaplastic gliomas
Source: Cancer Med. 2018 Aug 16;7(9):4273–83. doi: 10.1002/cam4.1672 (PMC6144143; doi:10.1002/cam4.1672)

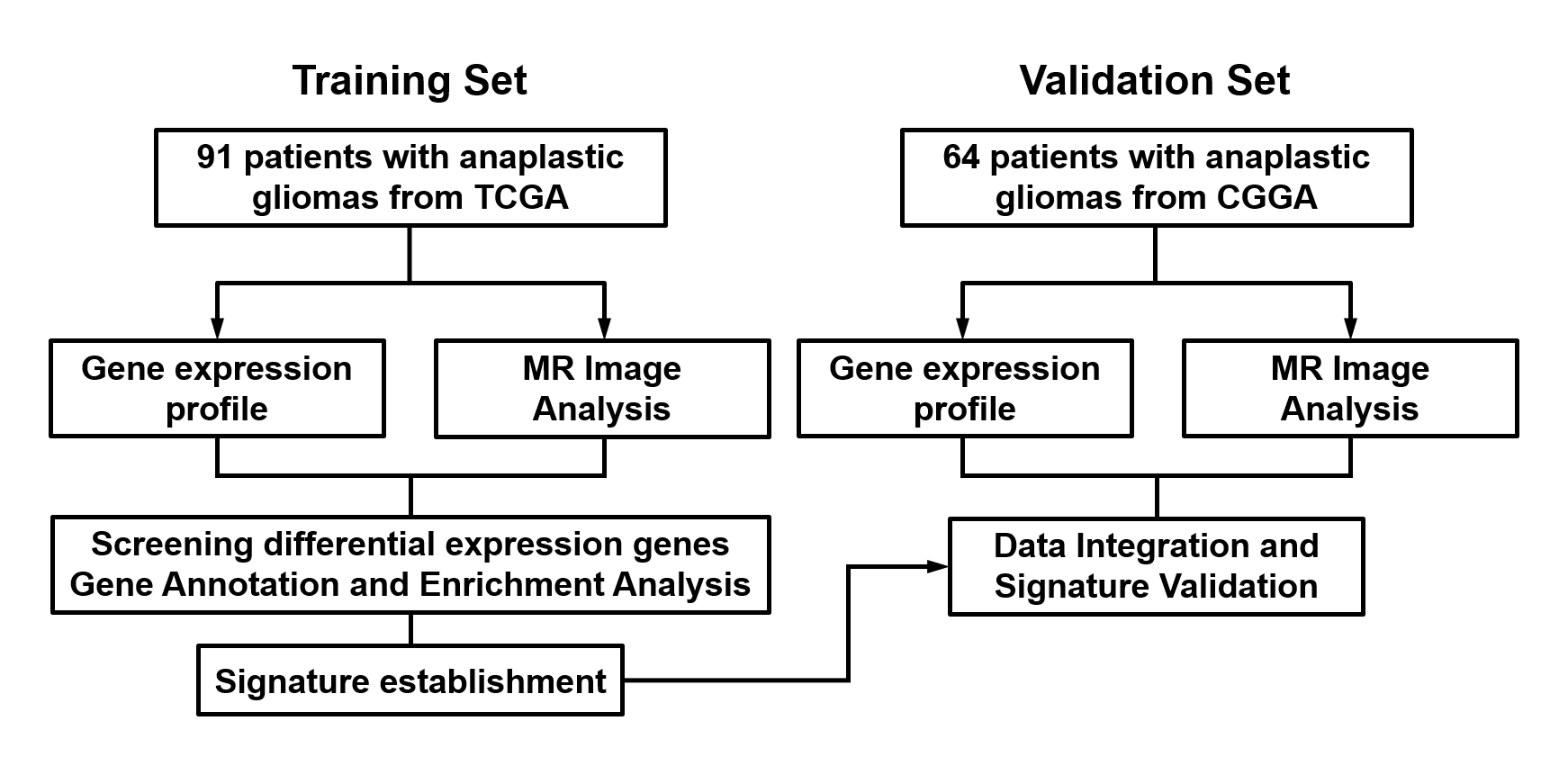

Supplement: Supplementary file 1 [file CAM4-7-4273-s001.tif]

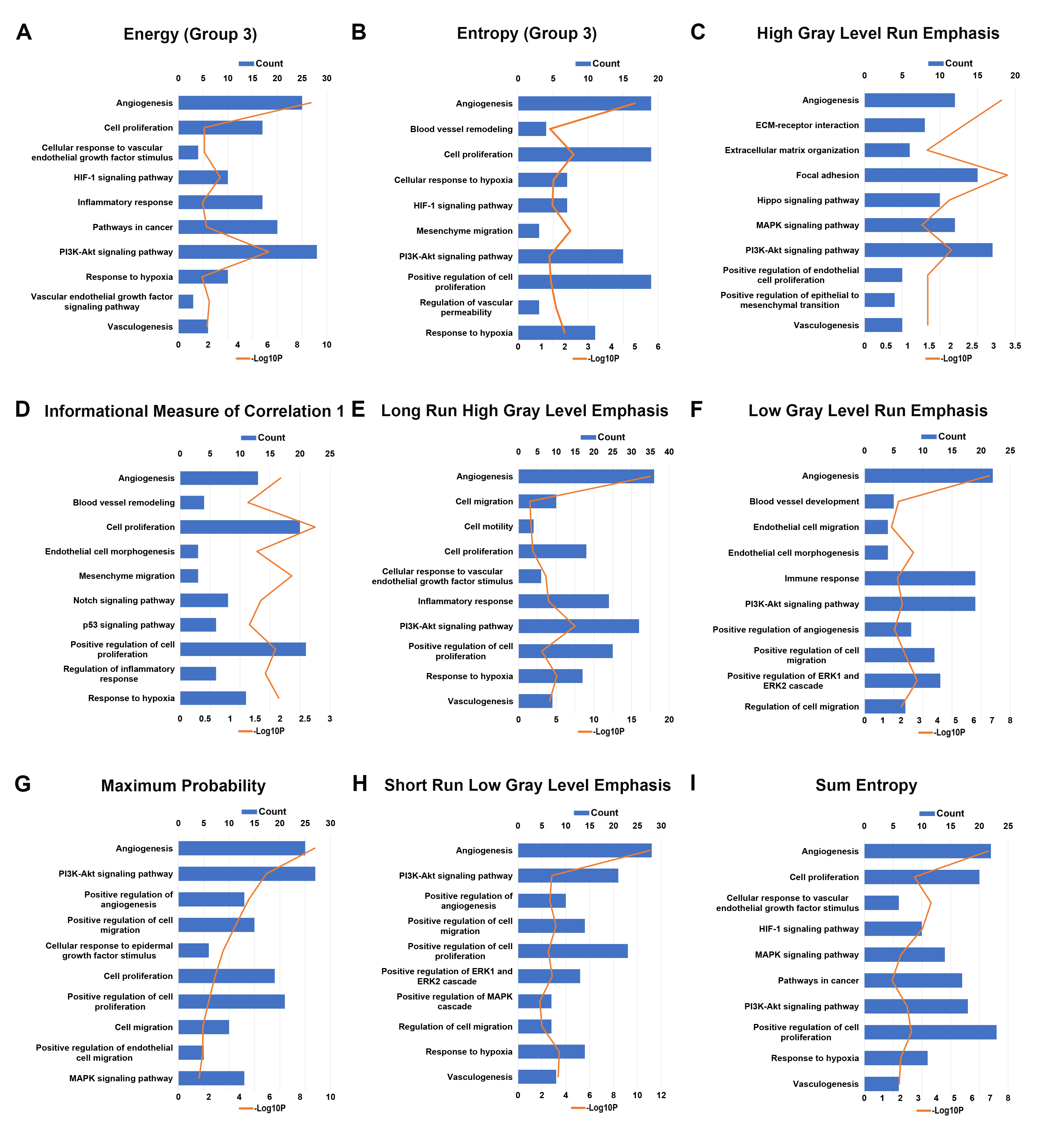

Supplement: Supplementary file 2 [file CAM4-7-4273-s002.tif]

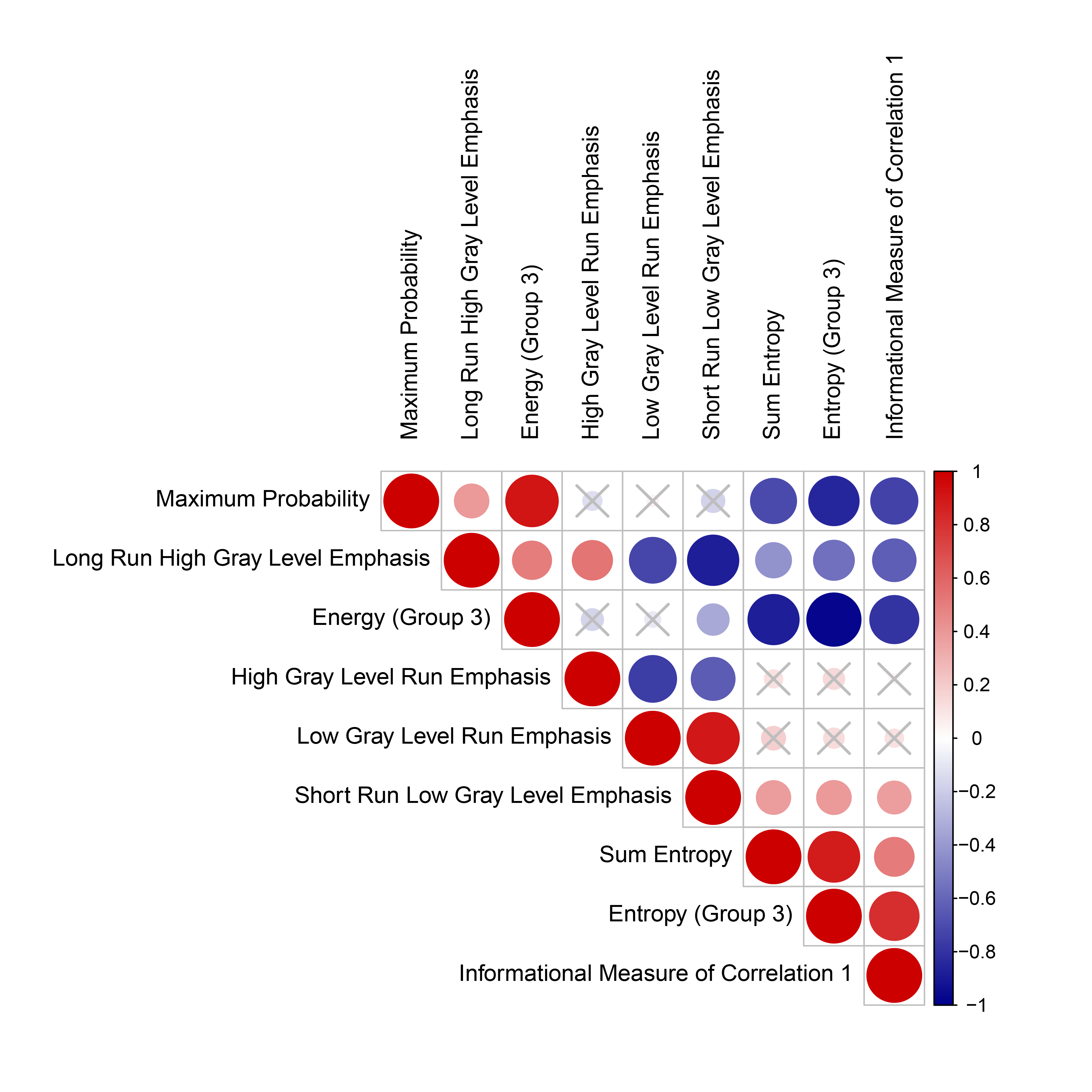

Supplement: Supplementary file 3 [file CAM4-7-4273-s003.tif]
