# Supplementary material for: Molecular profiles of tumor contrast enhancement: A radiogenomic analysis in anaplastic gliomas
Source: Cancer Med. 2018 Aug 16;7(9):4273–83. doi: 10.1002/cam4.1672 (PMC6144143; doi:10.1002/cam4.1672)
Supplement: Supplementary file 4 [file CAM4-7-4273-s004.docx]

**Supporting Information Table S1:** Gene sets enrichment analysis

| **Gene Sets** | **Name** | **Size** | **ES** | **NES** | **NOM p-val** |
| --- | --- | --- | --- | --- | --- |
| **Pathway datasets** | PID_LYMPH_ANGIOGENESIS_PATHWAY | 25 | 0.715 | 1.656 | 0.002 |
|  | REACTOME_EXTRACELLULAR_MATRIX_ORGANIZATION | 71 | 0.725 | 1.558 | 0.004 |
|  | REACTOME_METAL_ION_SLC_TRANSPORTERS | 19 | 0.672 | 1.603 | 0.006 |
|  | REACTOME_COLLAGEN_FORMATION | 58 | 0.733 | 1.536 | 0.006 |
|  | REACTOME_TIE2_SIGNALING | 16 | 0.68 | 1.639 | 0.008 |
|  | BIOCARTA_INTRINSIC_PATHWAY | 15 | 0.755 | 1.571 | 0.008 |
|  | NABA_BASEMENT_MEMBRANES | 39 | 0.668 | 1.487 | 0.014 |
|  | NABA_COLLAGENS | 42 | 0.737 | 1.494 | 0.014 |
|  | REACTOME_SYNTHESIS_OF_GLYCOSYLPHOSPHATIDYLINOSITOL_GPI | 17 | 0.56 | 1.751 | 0.018 |
|  | KEGG_ECM_RECEPTOR_INTERACTION | 78 | 0.667 | 1.529 | 0.02 |
|  | NABA_CORE_MATRISOME | 230 | 0.593 | 1.486 | 0.022 |
|  | BIOCARTA_SPRY_PATHWAY | 18 | 0.659 | 1.623 | 0.022 |
|  | PID_AVB3_INTEGRIN_PATHWAY | 74 | 0.648 | 1.533 | 0.023 |
|  | REACTOME_PPARA_ACTIVATES_GENE_EXPRESSION | 95 | 0.407 | 1.576 | 0.023 |
|  | PID_INTEGRIN3_PATHWAY | 39 | 0.688 | 1.452 | 0.031 |
|  | PID_EPHA2_FWD_PATHWAY | 18 | 0.539 | 1.599 | 0.031 |
|  | PID_INTEGRIN1_PATHWAY | 63 | 0.705 | 1.483 | 0.036 |
|  | PID_SYNDECAN_1_PATHWAY | 44 | 0.742 | 1.48 | 0.037 |
|  | PID_TCPTP_PATHWAY | 42 | 0.555 | 1.536 | 0.037 |
|  | KEGG_SMALL_CELL_LUNG_CANCER | 84 | 0.486 | 1.453 | 0.038 |
|  | KEGG_FOCAL_ADHESION | 189 | 0.503 | 1.462 | 0.041 |
|  | BIOCARTA_MTA3_PATHWAY | 17 | 0.666 | 1.456 | 0.045 |
|  | NABA_ECM_GLYCOPROTEINS | 160 | 0.546 | 1.394 | 0.046 |
|  | REACTOME_INTEGRIN_CELL_SURFACE_INTERACTIONS | 74 | 0.608 | 1.44 | 0.049 |
| **Biological Process** | G1_S_TRANSITION_OF_MITOTIC_CELL_CYCLE | 27 | 0.691 | 1.858 | 0 |
|  | I_KAPPAB_KINASE_NF_KAPPAB_CASCADE | 110 | 0.512 | 1.756 | 0 |
|  | IMMUNE_RESPONSE | 182 | 0.483 | 1.731 | 0 |
|  | REGULATION_OF_I_KAPPAB_KINASE_NF_KAPPAB_CASCADE | 91 | 0.518 | 1.719 | 0 |
|  | POSITIVE_REGULATION_OF_I_KAPPAB_KINASE_NF_KAPPAB_CASCADE | 86 | 0.519 | 1.704 | 0 |
|  | IMMUNE_SYSTEM_PROCESS | 260 | 0.435 | 1.599 | 0 |
|  | RESPONSE_TO_EXTERNAL_STIMULUS | 226 | 0.42 | 1.537 | 0 |
|  | ORGAN_DEVELOPMENT | 436 | 0.375 | 1.442 | 0 |
|  | DEFENSE_RESPONSE | 189 | 0.424 | 1.545 | 0.001 |
|  | RESPONSE_TO_WOUNDING | 141 | 0.447 | 1.554 | 0.003 |
|  | EPIDERMIS_DEVELOPMENT | 40 | 0.593 | 1.726 | 0.003 |
|  | POSITIVE_REGULATION_OF_CELL_PROLIFERATION | 126 | 0.428 | 1.47 | 0.004 |
|  | PROTEOLYSIS | 166 | 0.406 | 1.439 | 0.005 |
|  | ORGAN_MORPHOGENESIS | 111 | 0.432 | 1.481 | 0.007 |
|  | ECTODERM_DEVELOPMENT | 47 | 0.523 | 1.557 | 0.007 |
|  | T_CELL_ACTIVATION | 34 | 0.59 | 1.672 | 0.008 |
|  | EXCRETION | 20 | 0.644 | 1.617 | 0.008 |
|  | MITOTIC_SISTER_CHROMATID_SEGREGATION | 15 | 0.686 | 1.62 | 0.01 |
|  | REGULATION_OF_BODY_FLUID_LEVELS | 43 | 0.51 | 1.53 | 0.01 |
|  | ANATOMICAL_STRUCTURE_MORPHOGENESIS | 298 | 0.353 | 1.327 | 0.01 |
|  | RESPONSE_TO_STRESS | 442 | 0.341 | 1.32 | 0.011 |
|  | LEUKOCYTE_ACTIVATION | 55 | 0.493 | 1.511 | 0.011 |
|  | JAK_STAT_CASCADE | 26 | 0.599 | 1.614 | 0.012 |
|  | LOCOMOTORY_BEHAVIOR | 62 | 0.471 | 1.467 | 0.013 |
|  | CELL_PROLIFERATION_GO_0008283 | 438 | 0.334 | 1.284 | 0.013 |
|  | VASCULATURE_DEVELOPMENT | 46 | 0.516 | 1.542 | 0.013 |
|  | LYMPHOCYTE_ACTIVATION | 50 | 0.515 | 1.551 | 0.013 |
|  | POSITIVE_REGULATION_OF_IMMUNE_SYSTEM_PROCESS | 39 | 0.526 | 1.505 | 0.014 |
|  | INFLAMMATORY_RESPONSE | 94 | 0.438 | 1.455 | 0.017 |
|  | POSITIVE_REGULATION_OF_MULTICELLULAR_ORGANISMAL_PROCESS | 49 | 0.497 | 1.499 | 0.018 |
|  | POSITIVE_REGULATION_OF_DEVELOPMENTAL_PROCESS | 195 | 0.372 | 1.336 | 0.018 |
|  | EXTRACELLULAR_STRUCTURE_ORGANIZATION_AND_BIOGENESIS | 29 | 0.568 | 1.571 | 0.019 |
|  | REGULATION_OF_IMMUNE_SYSTEM_PROCESS | 51 | 0.506 | 1.537 | 0.019 |
|  | POSITIVE_REGULATION_OF_SIGNAL_TRANSDUCTION | 117 | 0.414 | 1.42 | 0.019 |
|  | HUMORAL_IMMUNE_RESPONSE | 24 | 0.599 | 1.588 | 0.02 |
|  | CELLULAR_CATION_HOMEOSTASIS | 75 | 0.447 | 1.45 | 0.02 |
|  | MITOTIC_CELL_CYCLE | 147 | 0.379 | 1.357 | 0.02 |
|  | CHROMOSOME_SEGREGATION | 31 | 0.567 | 1.564 | 0.021 |
|  | CELL_CYCLE_PROCESS | 177 | 0.378 | 1.348 | 0.023 |
|  | TISSUE_DEVELOPMENT | 89 | 0.427 | 1.415 | 0.024 |
|  | CELLULAR_DEFENSE_RESPONSE | 40 | 0.522 | 1.508 | 0.025 |
|  | FEMALE_PREGNANCY | 26 | 0.589 | 1.582 | 0.027 |
|  | SKELETAL_DEVELOPMENT | 81 | 0.424 | 1.398 | 0.03 |
|  | CATION_HOMEOSTASIS | 78 | 0.436 | 1.402 | 0.03 |
|  | ION_HOMEOSTASIS | 93 | 0.423 | 1.392 | 0.031 |
|  | MITOSIS | 77 | 0.429 | 1.381 | 0.032 |
|  | SISTER_CHROMATID_SEGREGATION | 16 | 0.652 | 1.575 | 0.032 |
|  | LEUKOCYTE_DIFFERENTIATION | 28 | 0.534 | 1.462 | 0.034 |
|  | CELL_CYCLE_PHASE | 155 | 0.373 | 1.316 | 0.039 |
|  | M_PHASE_OF_MITOTIC_CELL_CYCLE | 80 | 0.431 | 1.4 | 0.039 |
|  | POSITIVE_REGULATION_OF_T_CELL_ACTIVATION | 17 | 0.606 | 1.48 | 0.039 |
|  | REGULATION_OF_CELL_PROLIFERATION | 263 | 0.341 | 1.272 | 0.039 |
|  | MULTI_ORGANISM_PROCESS | 108 | 0.402 | 1.358 | 0.039 |
|  | IMMUNE_EFFECTOR_PROCESS | 27 | 0.532 | 1.439 | 0.039 |
|  | PHOSPHOINOSITIDE_MEDIATED_SIGNALING | 39 | 0.495 | 1.431 | 0.046 |
|  | G_PROTEIN_SIGNALING_COUPLED_TO_IP3_SECOND_MESSENGERPHOSPHOLIPASE_C_ACTIVATING | 36 | 0.508 | 1.439 | 0.046 |
|  | HEMOSTASIS | 37 | 0.506 | 1.43 | 0.047 |
|  | ANATOMICAL_STRUCTURE_FORMATION | 46 | 0.483 | 1.437 | 0.047 |
|  | PROTEIN_SECRETION | 26 | 0.55 | 1.459 | 0.048 |
|  | REGULATION_OF_T_CELL_ACTIVATION | 21 | 0.566 | 1.446 | 0.048 |
|  | CELLULAR_POLYSACCHARIDE_METABOLIC_PROCESS | 15 | 0.618 | 1.45 | 0.049 |
|  | APOPTOSIS_GO | 391 | 0.316 | 1.214 | 0.05 |
| **Molecular function** | POLYSACCHARIDE_BINDING | 29 | 0.722 | 1.964 | 0 |
|  | EXTRACELLULAR_MATRIX_STRUCTURAL_CONSTITUENT | 22 | 0.767 | 1.953 | 0 |
|  | GLYCOSAMINOGLYCAN_BINDING | 28 | 0.716 | 1.931 | 0 |
|  | PATTERN_BINDING | 34 | 0.663 | 1.91 | 0 |
|  | CARBOHYDRATE_BINDING | 53 | 0.578 | 1.768 | 0 |
|  | MOLECULAR_ADAPTOR_ACTIVITY | 43 | 0.552 | 1.636 | 0.002 |
|  | PROTEIN_BINDING_BRIDGING | 49 | 0.553 | 1.656 | 0.003 |
|  | SH3_SH2_ADAPTOR_ACTIVITY | 38 | 0.586 | 1.718 | 0.003 |
|  | PROTEASE_INHIBITOR_ACTIVITY | 21 | 0.664 | 1.744 | 0.003 |
|  | PEPTIDASE_ACTIVITY | 137 | 0.429 | 1.512 | 0.004 |
|  | ENZYME_INHIBITOR_ACTIVITY | 90 | 0.478 | 1.571 | 0.005 |
|  | HEPARIN_BINDING | 19 | 0.714 | 1.812 | 0.006 |
|  | EXOPEPTIDASE_ACTIVITY | 25 | 0.612 | 1.618 | 0.006 |
|  | SERINE_HYDROLASE_ACTIVITY | 33 | 0.529 | 1.503 | 0.021 |
|  | CYTOKINE_ACTIVITY | 58 | 0.459 | 1.436 | 0.025 |
|  | SULFURIC_ESTER_HYDROLASE_ACTIVITY | 15 | 0.66 | 1.55 | 0.03 |
|  | SERINE_TYPE_PEPTIDASE_ACTIVITY | 32 | 0.531 | 1.486 | 0.03 |
|  | LIPID_TRANSPORTER_ACTIVITY | 24 | 0.554 | 1.491 | 0.032 |
|  | HYDROLASE_ACTIVITY_ACTING_ON_ESTER_BONDS | 238 | 0.344 | 1.27 | 0.044 |
|  | SEQUENCE_SPECIFIC_DNA_BINDING | 47 | 0.459 | 1.391 | 0.045 |
|  | SERINE_TYPE_ENDOPEPTIDASE_ACTIVITY | 28 | 0.513 | 1.411 | 0.046 |
|  | ENDOPEPTIDASE_ACTIVITY | 91 | 0.402 | 1.334 | 0.047 |
|  | METALLOPEPTIDASE_ACTIVITY | 39 | 0.495 | 1.427 | 0.048 |
| **Cellular component** | PROTEINACEOUS_EXTRACELLULAR_MATRIX | 84 | 0.667 | 2.2 | 0 |
|  | EXTRACELLULAR_MATRIX | 85 | 0.664 | 2.163 | 0 |
|  | EXTRACELLULAR_MATRIX_PART | 51 | 0.683 | 2.086 | 0 |
|  | COLLAGEN | 23 | 0.761 | 1.993 | 0 |
|  | EXTRACELLULAR_REGION | 309 | 0.503 | 1.885 | 0 |
|  | EXTRACELLULAR_REGION_PART | 239 | 0.488 | 1.815 | 0 |
|  | CHROMOSOMEPERICENTRIC_REGION | 31 | 0.633 | 1.754 | 0.002 |
|  | BASEMENT_MEMBRANE | 33 | 0.623 | 1.736 | 0.006 |
|  | SPINDLE | 38 | 0.569 | 1.623 | 0.008 |
|  | INTEGRIN_COMPLEX | 17 | 0.681 | 1.637 | 0.009 |
|  | EXTRACELLULAR_SPACE | 159 | 0.389 | 1.382 | 0.014 |
|  | KINETOCHORE | 25 | 0.569 | 1.55 | 0.027 |
|  | SOLUBLE_FRACTION | 126 | 0.383 | 1.335 | 0.042 |
| **KEGG pathway** | KEGG_ECM_RECEPTOR_INTERACTION | 78 | 0.667 | 2.133 | 0 |
|  | KEGG_COMPLEMENT_AND_COAGULATION_CASCADES | 47 | 0.632 | 1.912 | 0 |
|  | KEGG_FOCAL_ADHESION | 189 | 0.503 | 1.837 | 0 |
|  | KEGG_JAK_STAT_SIGNALING_PATHWAY | 104 | 0.528 | 1.779 | 0 |
|  | KEGG_CYTOKINE_CYTOKINE_RECEPTOR_INTERACTION | 169 | 0.431 | 1.545 | 0.001 |
|  | KEGG_SMALL_CELL_LUNG_CANCER | 84 | 0.486 | 1.567 | 0.001 |
|  | KEGG_GRAFT_VERSUS_HOST_DISEASE | 28 | 0.596 | 1.619 | 0.006 |
|  | KEGG_HEMATOPOIETIC_CELL_LINEAGE | 60 | 0.506 | 1.577 | 0.008 |
|  | KEGG_ALLOGRAFT_REJECTION | 27 | 0.596 | 1.618 | 0.009 |
|  | KEGG_CELL_ADHESION_MOLECULES_CAMS | 117 | 0.427 | 1.456 | 0.009 |
|  | KEGG_AUTOIMMUNE_THYROID_DISEASE | 28 | 0.617 | 1.722 | 0.009 |
|  | KEGG_NATURAL_KILLER_CELL_MEDIATED_CYTOTOXICITY | 102 | 0.42 | 1.43 | 0.014 |
|  | KEGG_TRYPTOPHAN_METABOLISM | 33 | 0.546 | 1.521 | 0.018 |
|  | KEGG_PRIMARY_IMMUNODEFICIENCY | 27 | 0.544 | 1.448 | 0.039 |
|  | KEGG_ANTIGEN_PROCESSING_AND_PRESENTATION | 59 | 0.448 | 1.398 | 0.05 |
