# Supplementary material for: Molecular profiles of tumor contrast enhancement: A radiogenomic analysis in anaplastic gliomas
Source: Cancer Med. 2018 Aug 16;7(9):4273–83. doi: 10.1002/cam4.1672 (PMC6144143; doi:10.1002/cam4.1672)
Supplement: Supplementary file 5 [file CAM4-7-4273-s005.docx]

**Supporting Information Table S2:** Gene ontology and Kyoto Encyclopedia of Genes and Genomes pathways analyzed using the online Database for Annotation, Visualization, and Integrated Discovery.

| **Category** | **Term** | **Count** | **P-Value** | **Fold Enrichment** |
| --- | --- | --- | --- | --- |
| GOTERM_BP_FAT | GO:0030198~extracellular matrix organization | 24 | 1.32E-12 | 6.476859 |
| GOTERM_BP_FAT | GO:0043062~extracellular structure organization | 25 | 3.22E-09 | 4.304661 |
| GOTERM_BP_FAT | GO:0009611~response to wounding | 48 | 6.6E-09 | 2.541862 |
| GOTERM_BP_FAT | GO:0030199~collagen fibril organization | 10 | 4.47E-07 | 9.678066 |
| GOTERM_BP_FAT | GO:0031589~cell-substrate adhesion | 16 | 1.79E-06 | 4.582268 |
| GOTERM_BP_FAT | GO:0007155~cell adhesion | 51 | 1.9E-06 | 2.044837 |
| GOTERM_BP_FAT | GO:0022610~biological adhesion | 51 | 1.94E-06 | 2.04192 |
| GOTERM_BP_FAT | GO:0042060~wound healing | 22 | 4.41E-06 | 3.232778 |
| GOTERM_BP_FAT | GO:0007596~blood coagulation | 15 | 1.44E-05 | 4.12741 |
| GOTERM_BP_FAT | GO:0050817~coagulation | 15 | 1.44E-05 | 4.12741 |
| GOTERM_BP_FAT | GO:0007599~hemostasis | 15 | 2.79E-05 | 3.89811 |
| GOTERM_BP_FAT | GO:0050878~regulation of body fluid levels | 17 | 4.09E-05 | 3.383891 |
| GOTERM_BP_FAT | GO:0051605~protein maturation by peptide bond cleavage | 13 | 4.98E-05 | 4.242594 |
| GOTERM_BP_FAT | GO:0001501~skeletal system development | 27 | 7.46E-05 | 2.375525 |
| GOTERM_BP_FAT | GO:0000041~transition metal ion transport | 11 | 0.000194 | 4.348314 |
| GOTERM_BP_FAT | GO:0007160~cell-matrix adhesion | 12 | 0.000306 | 3.784232 |
| GOTERM_BP_FAT | GO:0051604~protein maturation | 14 | 0.000396 | 3.220733 |
| GOTERM_BP_FAT | GO:0006959~humoral immune response | 11 | 0.000469 | 3.907978 |
| GOTERM_BP_FAT | GO:0002253~activation of immune response | 12 | 0.000493 | 3.582943 |
| GOTERM_BP_FAT | GO:0016485~protein processing | 13 | 0.000625 | 3.257706 |
| GOTERM_BP_FAT | GO:0002541~activation of plasma proteins involved in acute inflammatory response | 8 | 0.00073 | 5.221654 |
| GOTERM_BP_FAT | GO:0048584~positive regulation of response to stimulus | 20 | 0.00079 | 2.378508 |
| GOTERM_BP_FAT | GO:0002449~lymphocyte mediated immunity | 10 | 0.000797 | 4.009484 |
| GOTERM_BP_FAT | GO:0006954~inflammatory response | 24 | 0.00138 | 2.072595 |
| GOTERM_BP_FAT | GO:0050778~positive regulation of immune response | 14 | 0.001994 | 2.709858 |
| GOTERM_BP_FAT | GO:0055066~di-, tri-valent inorganic cation homeostasis | 19 | 0.00229 | 2.231219 |
| GOTERM_BP_FAT | GO:0002526~acute inflammatory response | 11 | 0.002508 | 3.150309 |
| GOTERM_BP_FAT | GO:0002252~immune effector process | 13 | 0.002956 | 2.722859 |
| GOTERM_BP_FAT | GO:0034329~cell junction assembly | 7 | 0.003059 | 4.791823 |
| GOTERM_BP_FAT | GO:0007242~intracellular signaling cascade | 64 | 0.00313 | 1.430135 |
| GOTERM_BP_FAT | GO:0030005~cellular di-, tri-valent inorganic cation homeostasis | 18 | 0.003139 | 2.225529 |
| GOTERM_BP_FAT | GO:0043588~skin development | 6 | 0.00326 | 5.806839 |
| GOTERM_BP_FAT | GO:0002443~leukocyte mediated immunity | 10 | 0.003422 | 3.263534 |
| GOTERM_BP_FAT | GO:0006956~complement activation | 7 | 0.003464 | 4.677732 |
| GOTERM_BP_FAT | GO:0019724~B cell mediated immunity | 8 | 0.003533 | 4.009484 |
| GOTERM_BP_FAT | GO:0048545~response to steroid hormone stimulus | 16 | 0.003601 | 2.338866 |
| GOTERM_BP_FAT | GO:0034330~cell junction organization | 8 | 0.003908 | 3.939142 |
| GOTERM_BP_FAT | GO:0030003~cellular cation homeostasis | 19 | 0.004346 | 2.099454 |
| GOTERM_BP_FAT | GO:0044259~multicellular organismal macromolecule metabolic process | 6 | 0.0044 | 5.432205 |
| GOTERM_BP_FAT | GO:0010033~response to organic substance | 40 | 0.005899 | 1.557081 |
| GOTERM_BP_FAT | GO:0002250~adaptive immune response | 9 | 0.005914 | 3.280487 |
| GOTERM_BP_FAT | GO:0002460~adaptive immune response based on somatic recombination of immune receptors built from immunoglobulin superfamily domains | 9 | 0.005914 | 3.280487 |
| GOTERM_BP_FAT | GO:0009628~response to abiotic stimulus | 24 | 0.006461 | 1.830417 |
| GOTERM_BP_FAT | GO:0055080~cation homeostasis | 20 | 0.006851 | 1.962685 |
| GOTERM_BP_FAT | GO:0007243~protein kinase cascade | 24 | 0.006894 | 1.820523 |
| GOTERM_BP_FAT | GO:0001503~ossification | 11 | 0.007839 | 2.684611 |
| GOTERM_BP_FAT | GO:0007044~cell-substrate junction assembly | 5 | 0.008166 | 6.101389 |
| GOTERM_BP_FAT | GO:0006955~immune response | 38 | 0.0082 | 1.545685 |
| GOTERM_BP_FAT | GO:0043407~negative regulation of MAP kinase activity | 6 | 0.008444 | 4.677732 |
| GOTERM_BP_FAT | GO:0055072~iron ion homeostasis | 6 | 0.008444 | 4.677732 |
| GOTERM_BP_FAT | GO:0044236~multicellular organismal metabolic process | 6 | 0.009485 | 4.551306 |
| GOTERM_BP_FAT | GO:0045087~innate immune response | 12 | 0.010224 | 2.440556 |
| GOTERM_BP_FAT | GO:0031667~response to nutrient levels | 15 | 0.010815 | 2.137035 |
| GOTERM_BP_FAT | GO:0002684~positive regulation of immune system process | 17 | 0.011248 | 2.004742 |
| GOTERM_BP_FAT | GO:0032964~collagen biosynthetic process | 3 | 0.011747 | 16.83983 |
| GOTERM_BP_FAT | GO:0070482~response to oxygen levels | 12 | 0.011895 | 2.388629 |
| GOTERM_BP_FAT | GO:0060348~bone development | 11 | 0.012297 | 2.510002 |
| GOTERM_BP_FAT | GO:0051789~response to protein stimulus | 10 | 0.013931 | 2.623027 |
| GOTERM_BP_FAT | GO:0008544~epidermis development | 14 | 0.014385 | 2.135486 |
| GOTERM_BP_FAT | GO:0042110~T cell activation | 11 | 0.014386 | 2.45024 |
| GOTERM_BP_FAT | GO:0001568~blood vessel development | 17 | 0.014518 | 1.947464 |
| GOTERM_BP_FAT | GO:0001775~cell activation | 19 | 0.014586 | 1.858054 |
| GOTERM_BP_FAT | GO:0032963~collagen metabolic process | 5 | 0.016425 | 5.011855 |
| GOTERM_BP_FAT | GO:0045598~regulation of fat cell differentiation | 4 | 0.017713 | 7.016598 |
| GOTERM_BP_FAT | GO:0001944~vasculature development | 17 | 0.017858 | 1.900911 |
| GOTERM_BP_FAT | GO:0006826~iron ion transport | 5 | 0.018531 | 4.839033 |
| GOTERM_BP_FAT | GO:0006958~complement activation, classical pathway | 5 | 0.018531 | 4.839033 |
| GOTERM_BP_FAT | GO:0016477~cell migration | 18 | 0.02017 | 1.830417 |
| GOTERM_BP_FAT | GO:0051270~regulation of cell motion | 14 | 0.020559 | 2.035904 |
| GOTERM_BP_FAT | GO:0030149~sphingolipid catabolic process | 4 | 0.020952 | 6.603856 |
| GOTERM_BP_FAT | GO:0046466~membrane lipid catabolic process | 4 | 0.020952 | 6.603856 |
| GOTERM_BP_FAT | GO:0001666~response to hypoxia | 11 | 0.021248 | 2.303957 |
| GOTERM_BP_FAT | GO:0001558~regulation of cell growth | 14 | 0.021351 | 2.02541 |
| GOTERM_BP_FAT | GO:0030097~hemopoiesis | 16 | 0.021906 | 1.902806 |
| GOTERM_BP_FAT | GO:0009725~response to hormone stimulus | 22 | 0.022129 | 1.682454 |
| GOTERM_BP_FAT | GO:0002455~humoral immune response mediated by circulating immunoglobulin | 5 | 0.023227 | 4.526837 |
| GOTERM_BP_FAT | GO:0006879~cellular iron ion homeostasis | 5 | 0.023227 | 4.526837 |
| GOTERM_BP_FAT | GO:0034446~substrate adhesion-dependent cell spreading | 3 | 0.023529 | 12.02845 |
| GOTERM_BP_FAT | GO:0045599~negative regulation of fat cell differentiation | 3 | 0.023529 | 12.02845 |
| GOTERM_BP_FAT | GO:0048534~hemopoietic or lymphoid organ development | 17 | 0.023683 | 1.83511 |
| GOTERM_BP_FAT | GO:0046717~acid secretion | 4 | 0.024493 | 6.236976 |
| GOTERM_BP_FAT | GO:0006643~membrane lipid metabolic process | 8 | 0.024949 | 2.771989 |
| GOTERM_BP_FAT | GO:0007398~ectoderm development | 14 | 0.025659 | 1.97452 |
| GOTERM_BP_FAT | GO:0046649~lymphocyte activation | 14 | 0.025659 | 1.97452 |
| GOTERM_BP_FAT | GO:0009991~response to extracellular stimulus | 15 | 0.026011 | 1.913618 |
| GOTERM_BP_FAT | GO:0048870~cell motility | 19 | 0.026927 | 1.737008 |
| GOTERM_BP_FAT | GO:0051674~localization of cell | 19 | 0.026927 | 1.737008 |
| GOTERM_BP_FAT | GO:0009266~response to temperature stimulus | 8 | 0.028064 | 2.705194 |
| GOTERM_BP_FAT | GO:0050851~antigen receptor-mediated signaling pathway | 5 | 0.028583 | 4.252483 |
| GOTERM_BP_FAT | GO:0006952~defense response | 32 | 0.031646 | 1.460365 |
| GOTERM_BP_FAT | GO:0000188~inactivation of MAPK activity | 4 | 0.032479 | 5.613278 |
| GOTERM_BP_FAT | GO:0007626~locomotory behavior | 17 | 0.036399 | 1.741345 |
| GOTERM_BP_FAT | GO:0007229~integrin-mediated signaling pathway | 7 | 0.037886 | 2.806639 |
| GOTERM_BP_FAT | GO:0002520~immune system development | 17 | 0.038459 | 1.728727 |
| GOTERM_BP_FAT | GO:0001525~angiogenesis | 11 | 0.038533 | 2.086015 |
| GOTERM_BP_FAT | GO:0048514~blood vessel morphogenesis | 14 | 0.038963 | 1.862225 |
| GOTERM_BP_FAT | GO:0030334~regulation of cell migration | 12 | 0.039019 | 1.99288 |
| GOTERM_BP_FAT | GO:0006518~peptide metabolic process | 6 | 0.039463 | 3.177327 |
| GOTERM_BP_FAT | GO:0043086~negative regulation of catalytic activity | 17 | 0.039585 | 1.722486 |
| GOTERM_BP_FAT | GO:0006986~response to unfolded protein | 7 | 0.040194 | 2.767109 |
| GOTERM_BP_FAT | GO:0010038~response to metal ion | 10 | 0.0406 | 2.175689 |
| GOTERM_BP_FAT | GO:0019220~regulation of phosphate metabolic process | 26 | 0.040719 | 1.50459 |
| GOTERM_BP_FAT | GO:0051174~regulation of phosphorus metabolic process | 26 | 0.040719 | 1.50459 |
| GOTERM_BP_FAT | GO:0030595~leukocyte chemotaxis | 5 | 0.041328 | 3.792755 |
| GOTERM_BP_FAT | GO:0048286~lung alveolus development | 4 | 0.041653 | 5.10298 |
| GOTERM_BP_FAT | GO:0040012~regulation of locomotion | 13 | 0.041685 | 1.900328 |
| GOTERM_BP_FAT | GO:0007565~female pregnancy | 9 | 0.042254 | 2.296341 |
| GOTERM_BP_FAT | GO:0008360~regulation of cell shape | 6 | 0.042271 | 3.118488 |
| GOTERM_BP_FAT | GO:0016064~immunoglobulin mediated immune response | 6 | 0.042271 | 3.118488 |
| GOTERM_BP_FAT | GO:0002521~leukocyte differentiation | 10 | 0.044073 | 2.142473 |
| GOTERM_BP_FAT | GO:0042325~regulation of phosphorylation | 25 | 0.044873 | 1.505708 |
| GOTERM_BP_FAT | GO:0030001~metal ion transport | 25 | 0.045117 | 1.508946 |
| GOTERM_BP_FAT | GO:0006873~cellular ion homeostasis | 21 | 0.046396 | 1.57592 |
| GOTERM_BP_FAT | GO:0050852~T cell receptor signaling pathway | 4 | 0.046676 | 4.881111 |
| GOTERM_BP_FAT | GO:0008104~protein localization | 42 | 0.046711 | 1.336495 |
| GOTERM_BP_FAT | GO:0051216~cartilage development | 7 | 0.047646 | 2.654929 |
| GOTERM_BP_FAT | GO:0002429~immune response-activating cell surface receptor signaling pathway | 5 | 0.048726 | 3.598255 |
| GOTERM_BP_FAT | GO:0051781~positive regulation of cell division | 5 | 0.048726 | 3.598255 |
| GOTERM_BP_FAT | GO:0060326~cell chemotaxis | 5 | 0.048726 | 3.598255 |
| GOTERM_BP_FAT | GO:0015674~di-, tri-valent inorganic cation transport | 12 | 0.049777 | 1.913618 |
| GOTERM_CC_FAT | GO:0031012~extracellular matrix | 52 | 8.42E-18 | 4.0817 |
| GOTERM_CC_FAT | GO:0005578~proteinaceous extracellular matrix | 47 | 1.32E-15 | 3.97745 |
| GOTERM_CC_FAT | GO:0044421~extracellular region part | 84 | 1.17E-13 | 2.369544 |
| GOTERM_CC_FAT | GO:0005576~extracellular region | 135 | 7.24E-13 | 1.81884 |
| GOTERM_CC_FAT | GO:0044420~extracellular matrix part | 20 | 4.93E-08 | 4.629147 |
| GOTERM_CC_FAT | GO:0005604~basement membrane | 16 | 1.3E-07 | 5.554976 |
| GOTERM_CC_FAT | GO:0030141~secretory granule | 22 | 2.94E-06 | 3.30984 |
| GOTERM_CC_FAT | GO:0031982~vesicle | 50 | 3.23E-06 | 2.020933 |
| GOTERM_CC_FAT | GO:0031410~cytoplasmic vesicle | 48 | 5.1E-06 | 2.024711 |
| GOTERM_CC_FAT | GO:0045177~apical part of cell | 19 | 0.000109 | 2.874467 |
| GOTERM_CC_FAT | GO:0031988~membrane-bounded vesicle | 40 | 0.000134 | 1.907078 |
| GOTERM_CC_FAT | GO:0005581~collagen | 8 | 0.000242 | 6.189831 |
| GOTERM_CC_FAT | GO:0031983~vesicle lumen | 9 | 0.000243 | 5.29836 |
| GOTERM_CC_FAT | GO:0016023~cytoplasmic membrane-bounded vesicle | 38 | 0.000301 | 1.871017 |
| GOTERM_CC_FAT | GO:0016324~apical plasma membrane | 15 | 0.000389 | 3.054193 |
| GOTERM_CC_FAT | GO:0060205~cytoplasmic membrane-bounded vesicle lumen | 8 | 0.001036 | 4.923729 |
| GOTERM_CC_FAT | GO:0044459~plasma membrane part | 108 | 0.001144 | 1.327596 |
| GOTERM_CC_FAT | GO:0005615~extracellular space | 42 | 0.001476 | 1.660411 |
| GOTERM_CC_FAT | GO:0048471~perinuclear region of cytoplasm | 22 | 0.002303 | 2.06865 |
| GOTERM_CC_FAT | GO:0031093~platelet alpha granule lumen | 7 | 0.003645 | 4.623501 |
| GOTERM_CC_FAT | GO:0005588~collagen type V | 3 | 0.003966 | 27.08051 |
| GOTERM_CC_FAT | GO:0031091~platelet alpha granule | 8 | 0.004292 | 3.868644 |
| GOTERM_CC_FAT | GO:0005794~Golgi apparatus | 48 | 0.005421 | 1.49067 |
| GOTERM_CC_FAT | GO:0005583~fibrillar collagen | 4 | 0.008531 | 9.026836 |
| GOTERM_CC_FAT | GO:0030133~transport vesicle | 8 | 0.010536 | 3.282486 |
| GOTERM_CC_FAT | GO:0009898~internal side of plasma membrane | 21 | 0.013534 | 1.799654 |
| GOTERM_CC_FAT | GO:0005605~basal lamina | 4 | 0.023007 | 6.371884 |
| GOTERM_CC_FAT | GO:0005606~laminin-1 complex | 3 | 0.025161 | 11.60593 |
| GOTERM_CC_FAT | GO:0030935~sheet-forming collagen | 3 | 0.025161 | 11.60593 |
| GOTERM_CC_FAT | GO:0005886~plasma membrane | 159 | 0.030729 | 1.140006 |
| GOTERM_CC_FAT | GO:0009986~cell surface | 21 | 0.033179 | 1.634169 |
| GOTERM_CC_FAT | GO:0016323~basolateral plasma membrane | 14 | 0.037669 | 1.867621 |
| GOTERM_CC_FAT | GO:0043256~laminin complex | 3 | 0.041083 | 9.026836 |
| GOTERM_CC_FAT | GO:0005773~vacuole | 16 | 0.046471 | 1.719397 |
| GOTERM_CC_FAT | GO:0000323~lytic vacuole | 14 | 0.047909 | 1.796811 |
| GOTERM_CC_FAT | GO:0005764~lysosome | 14 | 0.047909 | 1.796811 |
| GOTERM_CC_FAT | GO:0009897~external side of plasma membrane | 12 | 0.049935 | 1.911565 |
| GOTERM_MF_FAT | GO:0005201~extracellular matrix structural constituent | 18 | 7.25E-09 | 5.869054 |
| GOTERM_MF_FAT | GO:0005518~collagen binding | 9 | 3.04E-05 | 7.010259 |
| GOTERM_MF_FAT | GO:0019838~growth factor binding | 13 | 0.000347 | 3.471747 |
| GOTERM_MF_FAT | GO:0030246~carbohydrate binding | 27 | 0.000388 | 2.138723 |
| GOTERM_MF_FAT | GO:0001871~pattern binding | 16 | 0.000391 | 2.913354 |
| GOTERM_MF_FAT | GO:0030247~polysaccharide binding | 16 | 0.000391 | 2.913354 |
| GOTERM_MF_FAT | GO:0005509~calcium ion binding | 52 | 0.000989 | 1.586653 |
| GOTERM_MF_FAT | GO:0005520~insulin-like growth factor binding | 6 | 0.001644 | 6.729849 |
| GOTERM_MF_FAT | GO:0004175~endopeptidase activity | 26 | 0.001966 | 1.944179 |
| GOTERM_MF_FAT | GO:0070011~peptidase activity, acting on L-amino acid peptides | 34 | 0.002274 | 1.736603 |
| GOTERM_MF_FAT | GO:0008484~sulfuric ester hydrolase activity | 5 | 0.002604 | 8.247364 |
| GOTERM_MF_FAT | GO:0008201~heparin binding | 11 | 0.003626 | 2.994674 |
| GOTERM_MF_FAT | GO:0005539~glycosaminoglycan binding | 13 | 0.004245 | 2.603811 |
| GOTERM_MF_FAT | GO:0008233~peptidase activity | 34 | 0.00456 | 1.660967 |
| GOTERM_MF_FAT | GO:0008237~metallopeptidase activity | 15 | 0.00583 | 2.298446 |
| GOTERM_MF_FAT | GO:0048407~platelet-derived growth factor binding | 4 | 0.005967 | 10.19674 |
| GOTERM_MF_FAT | GO:0004065~arylsulfatase activity | 4 | 0.007748 | 9.347012 |
| GOTERM_MF_FAT | GO:0005507~copper ion binding | 8 | 0.011132 | 3.251135 |
| GOTERM_MF_FAT | GO:0004857~enzyme inhibitor activity | 18 | 0.016557 | 1.869402 |
| GOTERM_MF_FAT | GO:0016715~oxidoreductase activity, acting on paired donors, with incorporation or reduction of molecular oxygen, reduced ascorbate as one donor, and incorporation of one atom of oxygen | 3 | 0.017233 | 14.02052 |
| GOTERM_MF_FAT | GO:0005178~integrin binding | 7 | 0.018026 | 3.326903 |
| GOTERM_MF_FAT | GO:0004252~serine-type endopeptidase activity | 12 | 0.021728 | 2.185016 |
| GOTERM_MF_FAT | GO:0008236~serine-type peptidase activity | 13 | 0.025402 | 2.047941 |
| GOTERM_MF_FAT | GO:0017171~serine hydrolase activity | 13 | 0.027401 | 2.025186 |
| GOTERM_MF_FAT | GO:0002020~protease binding | 4 | 0.028391 | 5.903376 |
| GOTERM_MF_FAT | GO:0042277~peptide binding | 14 | 0.029666 | 1.933865 |
| GOTERM_MF_FAT | GO:0004222~metalloendopeptidase activity | 9 | 0.031937 | 2.426628 |
| GOTERM_MF_FAT | GO:0004866~endopeptidase inhibitor activity | 11 | 0.034335 | 2.127251 |
| GOTERM_MF_FAT | GO:0030414~peptidase inhibitor activity | 11 | 0.046727 | 2.016022 |
| GOTERM_MF_FAT | GO:0005546~phosphatidylinositol-4,5-bisphosphate binding | 3 | 0.04706 | 8.412311 |
| KEGG_PATHWAY | hsa04512:ECM-receptor interaction | 20 | 3.46E-11 | 6.801766 |
| KEGG_PATHWAY | hsa04510:Focal adhesion | 24 | 3.24E-07 | 3.411035 |
| KEGG_PATHWAY | hsa04610:Complement and coagulation cascades | 10 | 0.000572 | 4.140205 |
| KEGG_PATHWAY | hsa05222:Small cell lung cancer | 10 | 0.002377 | 3.400883 |
| KEGG_PATHWAY | hsa04060:Cytokine-cytokine receptor interaction | 19 | 0.004093 | 2.071683 |
| KEGG_PATHWAY | hsa05412:Arrhythmogenic right ventricular cardiomyopathy (ARVC) | 8 | 0.015912 | 3.007096 |
| KEGG_PATHWAY | hsa05410:Hypertrophic cardiomyopathy (HCM) | 8 | 0.027723 | 2.688698 |
| KEGG_PATHWAY | hsa00531:Glycosaminoglycan degradation | 4 | 0.034778 | 5.441413 |
| KEGG_PATHWAY | hsa04810:Regulation of actin cytoskeleton | 14 | 0.035855 | 1.860204 |
| KEGG_PATHWAY | hsa00760:Nicotinate and nicotinamide metabolism | 4 | 0.049078 | 4.761236 |
