# Supplementary material for: Molecular profiles of tumor contrast enhancement: A radiogenomic analysis in anaplastic gliomas
Source: Cancer Med. 2018 Aug 16;7(9):4273–83. doi: 10.1002/cam4.1672 (PMC6144143; doi:10.1002/cam4.1672)
Supplement: Supplementary file 6 [file CAM4-7-4273-s006.docx]

**Supporting Information Table S3.** Brief description of 48 genes involved in the contrast enhancement signature

| **Symbol** | **Official Full Name** | **Gene Annotation** |
| --- | --- | --- |
| **CE Related Genes** | | |
| ABCC3 | ATP binding cassette subfamily C member 3 | Anion transmembrane transport |
| ALPK2 | Alpha kinase 2 | Protein phosphorylation |
| AOX1 | Aldehyde oxidase 1 | Inflammatory response |
| ARSF | Arylsulfatase F | Arylsulfatase activity |
| CDSN | Corneodesmosin | Cell adhesion |
| DNAH11 | Dynein axonemal heavy chain 11 | Microtubule motor activity |
| DOK7 | Docking protein 7 | Positive regulation of protein tyrosine kinase activity |
| DPP4 | Dipeptidyl-peptidase 4 | Positive regulation of cell proliferation |
| ERP27 | Endoplasmic reticulum protein 27 | Protein binding |
| F2RL2 | Coagulation factor II (thrombin) receptor-like 2 | Phosphatidylinositol phospholipase C activity |
| FBLN7 | Fibulin 7 | Cell adhesion |
| GALNT3 | Polypeptide N-acetylgalactosaminyltransferase 3 | O-glycan processing |
| GAS2L3 | Growth arrest specific 2 like 3 | Actin cytoskeleton organization |
| GDF15 | Growth differentiation factor 15 | SMAD protein signal transduction |
| HIST1H4J | Histone cluster 1, H4j | Beta-catenin-TCF complex assembly |
| HOXB3 | Homeobox B3 | Angiogenesis |
| HSPA7 | Heat shock protein family a (Hsp70) member 7 | ATP binding |
| IL13RA2 | Interleukin 13 receptor subunit alpha 2 | Cytokine receptor activity |
| IMPG2 | Interphotoreceptor matrix proteoglycan 2 | Extracellular matrix structural constituent |
| KMO | Kynurenine 3-monooxygenase (kynurenine 3-hydroxylase) | NAD(P)H oxidase activity |
| MACC1 | Metastasis associated in colon cancer 1 | Positive regulation of cell division |
| MAP1LC3C | Microtubule associated protein 1 light chain 3 gamma | Autophagosome assembly |
| MEOX2 | Mesenchyme homeobox 2 | Angiogenesis |
| MOXD1 | Monooxygenase, dbh-like 1 | Protein binding |
| NMUR1 | Neuromedin U receptor 1 | Activation of phospholipase C activity |
| OR1L8 | Olfactory receptor, family 1, subfamily l, member 8 | G-protein coupled receptor activity |
| POSTN | Periostin | Cell adhesion |
| RBM44 | Rna binding motif protein 44 | RNA binding |
| PLEK2 | Pleckstrin 2 | Actin cytoskeleton organization |
| SALL4 | Spalt-like transcription factor 4 | Protein binding |
| ESM1 | Endothelial cell specific molecule 1 | Angiogenesis |
| SCML4 | Sex comb on midleg-like 4 (drosophila) | DNA binding |
| SCN9A | Sodium channel, voltage gated, type IX alpha subunit | Inflammatory response |
| SLC47A2 | Solute carrier family 47 member 2 | Drug transmembrane transporter activity |
| TMEM26 | Transmembrane protein 26 | Integral component of membrane |
| TNFAIP6 | TNF alpha induced protein 6 | Inflammatory response |
| TPRG1 | Tumor protein p63 regulated 1 | Cytoplasm |
| WDR63 | WD repeat domain 63 | Protein binding |
| WNT16 | WNT family member 16 | WNT signaling pathway |
| LOC283314 |  |  |
| LOC285370 |  |  |
| LOC440896 |  |  |
| **nCE Related Genes** | | |
| SYNPO2L | Synaptopodin 2 like | Actin binding |
| WNT7B | WNT family member 7b | WNT signaling pathway |
| TNFSF18 | Tumor necrosis factor (ligand) superfamily, member 18 | Positive regulation of cell adhesion |
| RIPPLY2 | Ripply transcriptional repressor 2 | Notch signaling pathway |
| LRIT2 | Leucine-rich repeat, Ig-like and transmembrane domains 2 | Protein binding |
| F7 | Coagulation factor VII | Positive regulation of cell migration |
