# Supplementary material for: Molecular profiles of tumor contrast enhancement: A radiogenomic analysis in anaplastic gliomas
Source: Cancer Med. 2018 Aug 16;7(9):4273–83. doi: 10.1002/cam4.1672 (PMC6144143; doi:10.1002/cam4.1672)
Supplement: Supplementary file 7 [file CAM4-7-4273-s007.docx]

**Supporting Information Table S4:** Seven prognostic genes in LASSO-Cox regression model.

| **Symbol** | **HR** | **low95** | **high95** | **waldtest-P** |
| --- | --- | --- | --- | --- |
| POSTN | 1.309 | 1.167 | 1.468 | 4.38×10^-06^ |
| MAP1LC3C | 1.753 | 1.375 | 2.236 | 5.87×10^-06^ |
| TNFAIP6 | 1.921 | 1.441 | 2.561 | 8.40×10^-06^ |
| MEOX2 | 1.396 | 1.198 | 1.626 | 1.82×10^-05^ |
| GDF15 | 1.603 | 1.287 | 1.998 | 2.58×10^-05^ |
| TMEM26 | 1.665 | 1.275 | 2.173 | 1.78×10^-04^ |
| ABCC3 | 1.368 | 1.158 | 1.617 | 2.24×10^-04^ |
